# Chemical Equilibrium at the Tick–Host Feeding Interface:A Critical Examination of Biological Relevance in Hematophagous Behavior

**DOI:** 10.3389/fphys.2019.00530

**Published:** 2019-05-01

**Authors:** Ben J. Mans

**Affiliations:** ^1^Epidemiology, Parasites and Vectors, Agricultural Research Council-Onderstepoort Veterinary Research, Pretoria, South Africa; ^2^Department of Veterinary Tropical Diseases, University of Pretoria, Pretoria, South Africa; ^3^Department of Life and Consumer Sciences, University of South Africa, Pretoria, South Africa

**Keywords:** tick, host, feeding, affinity, equilibrium, function, relevance

## Abstract

Ticks secrete hundreds to thousands of proteins into the feeding site, that presumably all play important functions in the modulation of host defense mechanisms. The current review considers the assumption that tick proteins have functional relevance during feeding. The feeding site may be described as a closed system and could be treated as an ideal equilibrium system, thereby allowing modeling of tick–host interactions in an equilibrium state. In this equilibrium state, the concentration of host and tick proteins and their affinities will determine functional relevance at the tick–host interface. Using this approach, many characterized tick proteins may have functional relevant concentrations and affinities at the feeding site. Conversely, the feeding site is not an ideal closed system, but is dynamic and changing, leading to possible overestimation of tick protein concentration at the feeding site and consequently an overestimation of functional relevance. Ticks have evolved different possible strategies to deal with this dynamic environment and overcome the barrier that equilibrium kinetics poses to tick feeding. Even so, cognisance of the limitations that equilibrium binding place on deductions of functional relevance should serve as an important incentive to determine both the concentration and affinity of tick proteins proposed to be functional at the feeding site.

## Biological Activity at the Tick Feeding Interface

Biological function is central to the understanding of life and organismal biology. The context that function confers is exemplified in the frustration felt when a gene or protein of interest can only be annotated as a hypothetical protein with unknown function. Conversely, the ease of annotation by homology has made genome sequencing and assignment of function to orthologs an almost mundane task (Giles and Emes, 2017). Even so, biochemical characterization remains central to the elucidation and confirmation of new function and the understanding of functional mechanism. From a biochemical reductionist perspective, biological function may be explained within a structural mechanistic context as the action of a gene, surface, motif or residue that result in a chemical reaction, activation or inhibition of an enzyme or receptor, or even a physiological process such as blood clotting or platelet aggregation. The current review considers biological function at the tick–host interface from this reductionist perspective (Elgin, 2010). Even so, it was recently suggested that before or during evolution of new function by gene duplicates, and before negative selection maintain the adaptive advantage conferred by such new functions, new duplicates may exist in a state of undefined or non-optimized function, where protein expression is maintained while functional space is explored by random mutation: the playground hypothesis of neutral evolution (Mans et al., 2017). There is also an increasing recognition that most proteins may exhibit “promiscuous activity,” i.e., irrelevant secondary activities (Copley, 2015, 2017). In addition, given the possibility that a biochemical assay may yield results for a broad class of proteins with diverse functions, or even results without biological meaning, it raise the question whether any particular measured function is relevant within a biological or physiological context. The current review therefore also considers functional relevance at the tick–host interface from this perspective.

Sabbatani (1899), after reading Haycraft’s work on the anti-clotting effects of the oral secretions from leeches (Haycraft, 1884), made the first prescient deduction that all hematophagous organisms must secrete components that interfere or regulate host defenses, such as the hemostatic system. He tested this by showing that crude whole body tick extracts led to *in vitro* inhibition of blood clotting and that injecting this extract into various animals led to prolongation of blood coagulation *in vivo*. He also injected what may very well be the first fractionation of tick proteins to show that this purified preparation retained its inhibitory properties. Sabbatani’s deduction has been confirmed by extensive research into tick–host interactions that established a veritable pharmacopeia of bioactive components secreted by ticks during feeding (reviewed in Mans and Neitz, 2004a; Francischetti et al., 2009; Mans et al., 2016). However, a question that must have plagued him and remains relevant today, is whether *in vitro* and even *in vivo* observations can be causally linked with biological relevant activity at the feeding site, i.e., do what we measure in a test tube really function as a modulator of host defenses during feeding? The observation that ticks can cause paralysis in a host (Hovell, 1824 cited in Scott, 1921) and the presence of salivary glands in ticks (von Siebold and Stannius, 1854; Leydig, 1855; Heller, 1858), must have suggested that ticks can secrete substances into the host. Phenotypic changes in the host such as itching or ecchymosis after tick bite also suggested that ticks secrete substances into the host (Nuttall et al., 1908). Secretion and therefore presence would imply activity at the feeding site. However, the presence of toxic and anti-hemostatic molecules in tick eggs, but not salivary glands or saliva, showed that measurement of biological activity in crude extracts does not necessarily imply function at the tick–host interface (Hoeppli and Feng, 1933; de Meillon, 1942; Riek, 1957, 1959; Gregson, 1973; Neitz et al., 1981; Viljoen et al., 1985; Mans et al., 2004a). This implication was recognized soon after Sabbatani’s seminal study, when researchers extended his observations by proving that anti-hemostatic and toxic activities were present in salivary glands of ticks (Nuttall and Strickland, 1908; Cornwall and Patton, 1914; Ross, 1926; Hoeppli and Feng, 1933). It would take a number of years before anti-hemostatic and toxic activity could be showed to be secreted in tick saliva itself. This had to await chemical means, such as pilocarpine, or mechanical means, such as infra-red light, to stimulate salivation in order to obtain adequate quantities of salivary secretion for demonstration of biological activity (Howell, 1966; Tatchell, 1967; Neitz et al., 1969, 1978; Dickinson et al., 1976; Ribeiro et al., 1985, 1987; Ribeiro and Spielman, 1986; Ribeiro et al., 1991). However, as Tatchell (1967) indicated: salivary secretions obtained with exogenous stimulants should be treated with caution, since it is unclear whether such secretions represent the total saliva complement or even represent saliva, since cement is not found in such secretions. This may be a pertinent observation since cement may readily form during feeding on artificial membranes (Kröber and Geurin, 2007), arguing that induced salivation is not entirely the same as salivation during actual feeding.

Confirmation of secretion during feeding remains a crucial component of validation of biological relevance (Law et al., 1992). This may be achieved to various extents, by direct determination of the presence of a specific activity or molecule in saliva, or detection of host-derived antibodies generated against components secreted during feeding (Ribeiro et al., 1991; Oleaga-Pérez et al., 1994; Mulenga et al., 2003). Detection in the salivary glands or salivary gland extract (SGE) may be used as an indication of secretion, especially if a secretory peptide signal is present in the immature protein sequence (Nielsen, 2017). The latter have been extensively used to identify potential secretory components during transcriptome analysis (reviewed in Mans et al., 2016). However, secretion of some proteins without canonical signal peptides and non-salivary gland derived proteins via apocrine or alternative secretion has complicated the distinction of true and false positive secretory components (Mulenga et al., 2003; Díaz-Martín et al., 2013b; Oliveira et al., 2013; Tirloni et al., 2014, 2015), thereby also obscuring deduction of biological relevance (Mans et al., 2016). Not all salivary gland proteins with signal peptides are necessarily secreted during feeding (Nielsen, 2017), nor are all secretory proteins secreted at the same time, such as the case for hard ticks, that show differential expression over the course of several days of feeding (McSwain et al., 1982; Paesen et al., 1999; Wang et al., 2001b; de Castro et al., 2016, 2017; Kim et al., 2017; Perner et al., 2018). Transcriptome and proteome data also shows a weak correlation (Schwarz et al., 2014). While this may be ascribed to technical limitations in the proteomic and transcriptomic analysis of complex samples from non-model organisms, it further complicates the assessment of the final relevant concentration of protein present during feeding.

### The Equilibrium State and Functional Correlates

**FIGURE 1:**
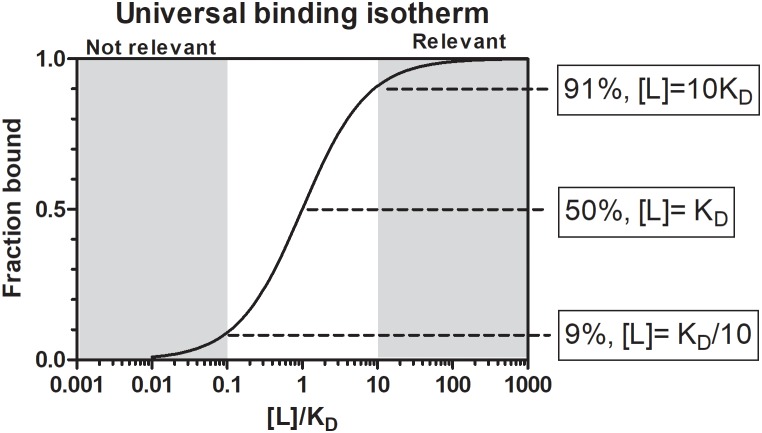
The universal binding isotherm express the relationship between receptor occupation by a ligand (fraction bound) and the equilibrium dissociation constant (*K*_D_) with regard to the concentration of ligand present for a 1:1 interaction at equilibrium. At a ligand concentration that equals the *K*_D_ (1*K*_D_), 50% of the receptor will be occupied. At a ligand concentration 10-fold more than the *K*_D_ (10*K*_D_), 91% of the receptor will be occupied. Above this concentration a given ligand should be physiologically significant. At a ligand concentration 10-fold less than the *K*_D_ (*K*_D_/10) only 9% of the receptor will be occupied. Below this concentration physiological significance should be suspect. Between 10*K*_D_ and *K*_D_/10 a gray area exist where physiological significance will depend on environmental factors.

Le Chatelier’s principle states that an equilibrium system will tend to counteract changes to the system to maintain its equilibrium. In biological systems this is overcome by enzymes that create intermediate states to catalyze non-reversible chemical reactions. In the case of receptor-ligand or protein-inhibitor binding, the affinity of the receptor for the ligand and the relative receptor and ligand concentrations determines the bound state at equilibrium (Figure 1). The affinity between molecules is expressed as the equilibrium dissociation constant (*K*_D_) that range for most biological systems from the fM to mM range (Smith et al., 2012). The *K*_D_ is the ligand concentration at which 50% of the receptor is occupied at equilibrium (Figure 1). At a ligand concentration 10-fold the *K*_D_, a receptor would be 91% saturated and above this concentration almost all of the receptor will be occupied. However, at a ligand concentration 10-fold less than the *K*_D_, only 9% of the receptor would be occupied by the ligand and below this concentration essentially no binding occurs. At concentrations of the ligand that is equal to its *K*_D_, only 50% of the receptor will be occupied if the stoichiometry of binding is 1:1. The *K*_D_ is therefore also considered to reflect a physiological relevant concentration (Sears et al., 2007). Concentrations of salivary proteins at the feeding site would therefore be physiologically relevant only at concentrations equal or exceeding their affinities for their targets by 10-fold or more if 50–100% receptor occupancy is necessary for effective inhibition. The *K*_i_ (inhibitor constant) is determined for inhibitors of enzyme active sites and reflect the concentration necessary to reduce enzyme activity by half. It is generally used in the characterization of inhibitors using enzyme kinetics and depends on both enzyme affinity for substrate and inhibitor affinity for enzyme. Even so, it is similar to the *K*_D_ and should produce similar values when enzyme activity is measured at physiological concentrations. Another measurement analogous to the *K*_D_ is the IC_50_ value of an inhibitor. The IC_50_ value (half-maximal inhibitory concentration) is the concentration at which 50% inhibition of function is observed. This is generally determined for complex processes such as inhibition of platelet aggregation, blood clotting, or cell migration. This value would ultimately depend on the concentration of receptor or number of cells used in an assay, but would be close to the *K*_D_ if physiologically relevant receptor or cell concentrations were used. Another measure that may indicate physiological relevance is the stoichiometric inhibition ratio (SI). This ratio indicate physiological relevance when an inhibitor interact with an enzyme or receptor close to equimolar ratios, since this imply high affinity, so that for example an SI = 2 imply saturation of receptor at 2*K*_D_. This may be seen for suicide inhibitors that bind irreversible to the enzyme active site. In enzyme kinetics the *K*_m_ (Michaelis constant) is the substrate concentration that allows an enzyme to attain half *V*_max_. *V*_max_ is the maximum reaction rate of the enzyme when saturated with substrate. While the *K*_m_ is dependent on rate constants rather than ligand concentration it is also an indication of affinity and provide an estimate of relevant concentrations of substrates where an enzyme will function. For example, if a given agonist functions at concentrations 10-fold lower than the *K*_m_, the enzyme will not be able to effectively remove this agonist from the system and neutralize its effect. The *K*_D_, *K*_i_, IC_50_, SI, and *K*_m_ are therefore all useful to assess the physiological relevance of any biological activity. The reader is referred to Kuriyan et al. (2013) for a general treatment of protein-ligand affinity.

Another measure of potential functional activity is the amount of salivary gland equivalents that may have a measurable impact or functionality. For example, ∼0.125 salivary gland equivalents from the tick *Ornithodoros kalahariensis* (previously *Ornithodoros savignyi*, Bakkes et al., 2018), was able to increase clotting time for the APTT test by 400%, 0.4% of a salivary gland could inhibit fXa ∼100 and 0.07% could inhibit thrombin by ∼100% (Gaspar et al., 1995). As such, any function that may be measured from the equivalent of 2 salivary glands (∼1 tick) or less that has an appreciable effect on some function may indicate the presence of relevant functionality. Even so, if characterizing crude extracts, it should be considered that the sum of multiple functions may be measured and that individual activities may be much less.

**FIGURE 2:**
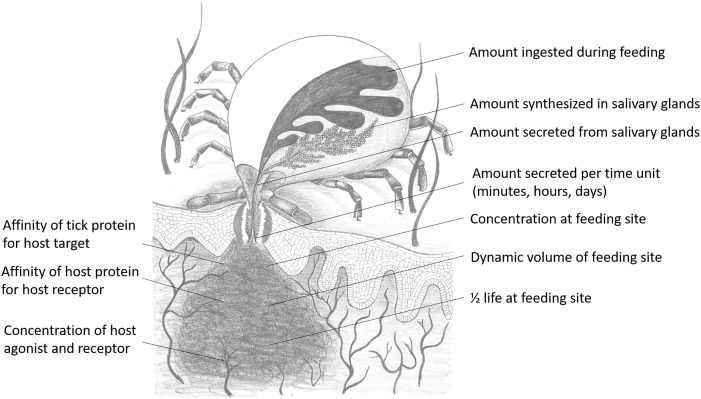
Parameters that influence functional relevance at the feeding site for any salivary gland protein secreted during feeding. Drawing courtesy of Ronel Pienaar.

Whether a molecule will have relevant biological activity at the feeding site depends on their affinities for their respective ligands or receptors and whether they are secreted at relevant biological concentrations. In terms of chemical equilibrium, this implies that inhibitors or kratagonists (dealt with below) needs to be present at the feeding site at higher concentrations than the *K*_D_ for their respective receptors or ligands. At concentrations below the *K*_D_ little or no binding will occur and inhibitors may not be effective or physiologically relevant. In addition to satisfying concentration requirements, competition between host-derived substrates, ligands or receptors and tick-derived inhibitors, enzymes or scavengers for activating biomolecules will determine whether potential host-modulatory molecules may be biologically significant. This is essentially determined by the comparative affinities of host vs. tick-derived receptor-ligand interaction. Again, the biological relevance of host-derived agonists will be determined by their active concentrations at the feeding site. Effective concentration may also be determined by protein turnover or half-life at the feeding site. This will depend on secretion into the feeding site, concentration and sequestration at the feeding site and protein stability. In the case of secretory proteins, most have disulphide bonds that increase their stability (Mans et al., 2016), contributing toward their effective concentrations. Modulation of host defenses at the tick–host interface therefore depends on interplay between these factors, with the implication that tick-derived antagonists need to be present at higher concentrations than host-derived agonists and have higher affinities for their shared targets (Figure 2).

### The Feeding Site: Effective Volume Determines Functionality

The equilibrium state is an ideal closed state and the feeding site may be considered as such. However, while the feeding site may have a defined volume approximating a closed state, the feeding site itself may be dynamic with constant changes in concentration of both host and tick proteins. Ticks ingests blood meal and salivary components, since ticks alternately salivate and ingest blood meal (Arthur, 1970; Kemp et al., 1982), thereby removing both volume and active components from the feeding site. These repetitive doses of saliva, alternating by sucking events may be considered to be independent equilibrium events and in such a case may suggest that estimates of concentration at the feeding site may be lower than described below. Tick-derived molecules may also be removed into the general systemic system of the host or the general area around the feeding site, as evidenced by the systemic effects seen during paralysis and tick toxicoses (Mans et al., 2004a). Influx of blood or lymph into the feeding site may also dilute tick-derived proteins as seen in the case of edema (Kemp et al., 1982). As such, while the feeding site itself has been described as a hematoma or cavity that seems self-contained, the site itself may be much more dynamic than a defined cavity of blood.

A critical question remains as to what extent the feeding site may be reduced to a biochemical reaction in a test tube, i.e., can we measure the feeding site, calculate the feeding site volume and determine the amount of saliva secreted by the tick to derive estimations of biological concentrations at the feeding site? However, the feeding site is a complex and dynamic environment that changes constantly as determined by both the tick and the host (Nuttall, 2019). For example, ticks secrete enzymes that degrade the extracellular matrix over time, increasing the feeding site volume, while host wound healing responses will tend to counteract an increase in feeding site volume (Wikel, 2017). On the other hand, host inflammatory responses such as edema and cellular infiltration will tend to increase the feeding site volume, while hard ticks actively secrete excess blood-meal derived water back into the host, which will further contribute to feeding site volume, dilution and drainage of salivary proteins. As such, feeding site volumes have been conservatively estimated at 10–50 μl (Mans et al., 2008a,b). However, feeding site volume differs between larvae, nymphs and adults, whether natural or secondary hosts are parasitized and whether hosts are naïve or immune (Tatchell and Moorhouse, 1968; Brown and Knapp, 1980a,b; Brown et al., 1983). Natural hosts tend to present smaller feeding lesions, while secondary hosts can produce large lesions due to inflammatory responses (Brown and Knapp, 1980a,b; Brown et al., 1983).

### Protein Concentration at the Feeding Site and Functionally Suspect Proteins

**FIGURE 3:**
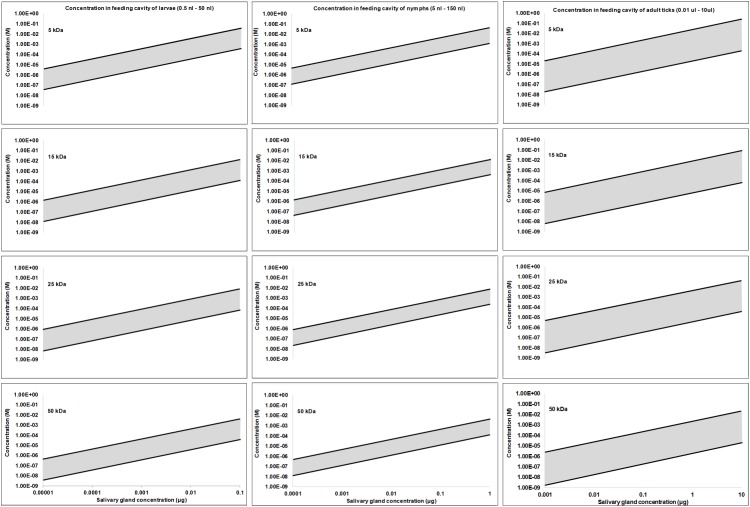
Protein concentration estimates at the feeding site for larvae, nymphs and adults. Feeding sites volumes were estimated from Brown and Knapp (1980a; 1980b) and salivary concentrations were simulated over a 10,000-fold range assuming a maximum concentration of 10 μg in adults, 1 μg in nymphs and 0.1 μg in larvae.

It may be estimated what concentrations can be expected for the average secreted protein at the feeding site, by asking what the highest protein concentration may be. In this regard, the lipocalins are known to be some of the most abundant proteins synthesized in tick salivary glands (Mans, 2011; Mans et al., 2017). The TSGP1-4 lipocalins from the soft tick *O. kalahariensis* has been characterized in detail. They each make up ∼5% of the total soluble salivary gland protein, comprising ∼20% of the total soluble protein content (Mans et al., 2001; Mans and Neitz, 2004b). In this case the total protein that may be secreted by a tick during a feeding event comprise ∼6 μg protein with molecular masses of ∼15 kDa each. Similarly, the savignygrins (Mr ∼ 7 kDa) also make up ∼3% of the total salivary gland protein with ∼4 μg of protein secreted during a feeding event (Mans et al., 2002b). In the case of the lipocalins from the soft ticks *Argas monolakensis*, the histamine binding protein AM-10 (Mr ∼ 16 kDa) comprise ∼23% of the total soluble protein (Mans et al., 2008b,c), and in this case equates with 4.6 μg of protein secreted during feeding. These concentrations are likely to be the highest estimates, since it is known that hard ticks secrete ∼30–300 times less protein than soft ticks in their saliva at any given feeding stage (Ribeiro, 1987; Dharampaul et al., 1993). The upper limit for any given protein in the adult tick salivary gland may thus be assumed to be ∼10 μg with a dynamic protein concentration range of 10,000-fold (1 ng–10 μg). If it is also assumed that nymphs have 10-fold and larvae 100-fold lower salivary concentrations than adults some interesting observations may be made regarding feeding site concentrations. For the purpose of the current study, the feeding site volumes were estimated from the studies of Brown and Knapp (1980a,b) with feeding cavities for larvae from 0.1 to 50 nl, nymphs from 5 to 150 nl, and adults from 10 nl to 10 μl. Using these assumptions concentration fluctuation at the feeding site can be estimated at various salivary gland protein concentrations and molecular masses of proteins. Under these assumptions the concentrations at the feeding site do not differ extensively between larvae, nymphs or adults, even though feeding site volume and effective salivary gland concentrations may differ (Figure 3). Allowing for the concentration ranges estimated, maximum concentrations at the feeding site may range from nM to mM, and may even range from nM to μM for salivary gland concentrations such as 0.1, 0.01, or 0.001 μg for adults, nymphs, or larvae, respectively, that may be considered to be more representative of the average protein concentration. These concentrations may be an over estimation since the dynamic nature of the feeding site and the rate at which protein is secreted may never approximate these total estimated concentrations.

**FIGURE 4:**
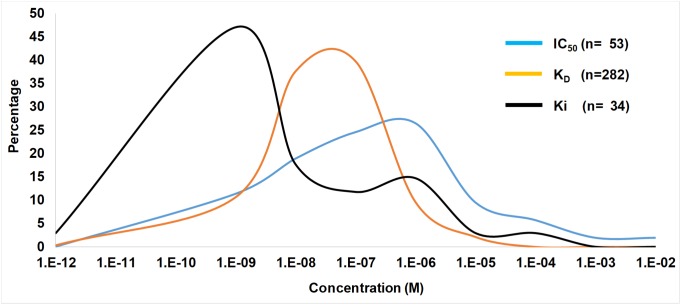
Ranges of IC_50_, *K*_D_, and *K*_i_ values from the tick literature. Indicated are frequency distribution curves of values obtained from Table 1 and references therein.

In ticks the IC_50_, *K*_D_, and *K*_i_ values range from pM–mM and falls within the concentration expected at the feeding site (Figure 3, 4). IC_50_ values generally tend to range from 10 nM to 10 μM (Figure 2). Conversely, *K*_D_ values range from 1 nM to 1 μM, while *K*_i_ values range from 10 pM to 10 nM. The higher IC_50_ values is probably due to the use of *in vitro* assays that measure complex reactions such as platelet aggregation, blood clotting, complement or cell migration. These systems do not necessarily represent true *in vivo* conditions and may be artificial to some extent, i.e., much higher concentrations of agonists or cell numbers are needed for observation than what is found under physiological conditions. The *K*_D_ values may represent true affinities, while the *K*_i_ values are dependent on the enzyme and substrate concentrations used, and the lower pM values may be due to the availability of chromogenic and fluorogenic substrates that enable sensitive measurement of enzyme activity. In this case, some of the affinities may be overestimated.

A number of tick proteins characterized have affinities, inhibition constants or IC_50_ values in the nM or pM range, which would probably be functional at the feeding site (Figure 4 and Table 1). For some, concentrations at the feeding site may be estimated based on yields of purified inhibitor and these are generally in the same range as the affinities or higher, suggesting biological relevant concentrations at the feeding site (Table 1). For a number of proteins, estimates of concentrations at the feeding site is not available and the low nM to pM range of their *K*_D_, *K*_i_, or IC_50_ values is the only indication of functional relevance. A number of proteins also present extraordinary high concentration estimates at the feeding sites (>1 M), which may indicate that these proteins may not have been completely purified at the time of their characterization, or may reflect that they derived from whole body extracts and not tick salivary glands, which may suggest that these inhibitors originate from multiple organs (Ibrahim et al., 2001a,b). In many cases, the concentration of inhibitors that may occur at the feeding site has not been determined (Table 1). This will remain a major impediment in the assessment of functional relevance at the feeding site, since we are probably over rather than underestimating feeding site concentrations. To further consider functional relevance it is necessary to unpack the salivary gland repertoire into its functional modalities.

**Table 1:** Tick inhibitors of host defenses considered to have relevant physiological function.

Protein	Tick	Target	Affinity (*K*_D_, *K*_i_, *K*_m_, IC_50_)	Concentration (Minimum–Maximum)	References
Apyrase	*Ixodes scapularis*	ATP/ADP	N/A	Activity in saliva	Ribeiro et al., 1985
Apyrase	*Ornithodoros moubata*	ATP/ADP	N/A	Activity in saliva	Ribeiro et al., 1991
Apyrase	*Ornithodoros kalahariensis*	ATP/ADP	*K*_m_ ~ 1 mM	Activity in saliva 1 μM–1 mM	Mans, 1997; Mans et al., 1998b
Ir-CPI	*Ixodes ricinus*	fXIIa, fXIa, Plasmin	*K*_D_ ~ 16–38 nM	Unknown	Decrem et al., 2009
PAI	*Ixodes sinensis*	Platelets	IC_50_ ~ 250 nM	Unknown	Liu et al., 2005
Savignygrin	*Ornithodoros kalahariensis*	α_IIb_β_3_	IC_50_ ~ 130 nM; *K*_D_ ~ 60 nM	55 μM–57 mM	Mans et al., 2002b; Mans and Neitz, 2004c
Monogrin	*Argas monolakensis*	α_IIb_β_3_	IC_50_ ~ 150 nM	10 μM–10 mM	Mans et al., 2008a
Variabilin	*Dermacentor variabilis*	α_IIb_β_3_	IC_50_ ~ 150 nM	2.7 μM–2.7 mM	Wang et al., 1996
Disagregin	*Ornithodoros moubata*	α_IIb_β_3_	IC_50_ ~ 104 nM; *K*_D_ ~ 40 nM	20 μM–20 μM	Karczewski et al., 1994
YY-39	*Ixodes scapularis*	α_IIb_β_3_	IC_50_ ~ 4–20 μM	Unknown	Tang et al., 2015
TAI	*Ornithodoros moubata*	α_2_β_1_	IC_50_ ~ 5–8 nM; *K*_D_ ~ 40 nM	50 nM–50 μM	Karczewski et al., 1995
Moubatin	*Ornithodoros moubata*	TXA_2_	IC_50_ ~ 50 nM *K*_D_ ~ 24 nM	1.5 μM–1.5 mM	Waxman and Connolly, 1993; Mans and Ribeiro, 2008b
TSGP2	*Ornithodoros kalahariensis*	LTB_4_	*K*_D_ ~ 18 nM	38 μM–38 mM	Mans et al., 2001; Mans and Ribeiro, 2008b
TSGP3	*Ornithodoros kalahariensis*	TXA_2_; LTB_4_	*K*_D_ ~ 5–21 nM	38 μM–38 mM	Mans et al., 2001; Mans and Ribeiro, 2008b
Ir-LBP	*Ixodes ricinus*	LTB_4_	*K*_D_ ~ 0.5 nM	Detected in saliva	Beaufays et al., 2008
TSGP4	*Ornithodoros kalahariensis*	LTC_4_	*K*_D_ < 2 nM	35 μM–35 mM	Mans et al., 2001; Mans and Ribeiro, 2008a
AM-33	*Argas monolakensis*	LTC_4_	*K*_D_ ~ 2 nM	3.5 μM–3.5 mM	Mans et al., 2008c; Mans and Ribeiro, 2008a
TSGP1	*Ornithodoros kalahariensis*	Histamine Serotonin	*K*_D_ < 3 nM; *K*_D_ ~ 6 nM	32 μM–32 mM	Mans et al., 2001, 2008b
OP-3	*Ornithodoros parkeri*	Histamine Serotonin	*K*_D_ ~ 106 nM; *K*_D_ ~ 116 nM	Detected in SGE	Francischetti et al., 2008 Mans and Ribeiro, 2008b
OtLip	*Ornithodoros turicata*	Histamine	Unknown	Unknown	Neelakanta et al., 2018
Monomine	*Argas monolakensis*	Histamine	*K*_D_ ~ 7 nM	29 μM–28 mM	Mans et al., 2008b,c
Monotonin	*Argas monolakensis*	Serotonin	*K*_D_ < 2 nM	10 μM–10 mM	Mans et al., 2008b,c
Is-14	*Ixodes scapularis*	Histamine Serotonin	*K*_D_ ~ 427 nM; *K*_D_ < 2	Unknown	Mans et al., 2008c
Is-15	*Ixodes scapularis*	Histamine Serotonin	*K*_D_ ~ 746 nM; *K*_D_ < 2	Unknown	Mans et al., 2008c
HBP1-3	*Rhipicephalus appendiculatus*	Histamine	*K*_D_ ~ 1–18 nM	Detected in SGE	Paesen et al., 1999
SHBP	*Dermacentor reticulatus*	Histamine Serotonin	*K*_D_ ~ 1–2 nM *K*_D_ ~ 0.6 nM	Unknown	Sangamnatdej et al., 2002
Savignin	*Ornithodoros kalahariensis*	Thrombin	*K*_i_ ~ 5 pM	850 nM–1 mM	Nienaber et al., 1999
P5	*Hyalomma dromedarii*	Thrombin	*K*_i_ ~ 500 nM	30 μM–30 mM	Ibrahim and Masoud, 2018
Sculptin	*Amblyomma sculptum*	Thrombin	IC_50_ ~ 2 pM *K*_i_ ~ 18 pM;	Unknown	Iqbal et al., 2017
Avathrin	*Amblyomma variegatum*	Thrombin	IC_50_ ~ 7 nM *K*_i_ ~ 545 pM	Unknown	Iyer et al., 2017
Boophilin	*Rhipicephalus microplus*	Thrombin	IC_50_ ~ 1 μM *K*_i_ ~ 101, 0.5–2 nM *K*_D_ ~ 116 nM	Unknown	Macedo-Ribeiro et al., 2008; Soares et al., 2012; Assumpção et al., 2016
RmS-15	*Rhipicephalus microplus*	Thrombin	SI ~ 2	Unknown	Rodriguez-Valle et al., 2015; Xu T. et al., 2016
Hyalomin-1	*Hyalomma rufipes*	Thrombin	*K*_i_ ~ 12 nM *K*_D_ ~ 19 nM	Unknown	Jablonka et al., 2015
rIxscS-1E1	*Ixodes scapularis*	Thrombin	SI ~ 4	Unknown	Ibelli et al., 2014
Monobin	*Argas monolakensis*	Thrombin	*K*_i_ ~ 6 pM	400 nM–400 μM	Mans et al., 2008a
Chimadanin	*Haemaphysalis longicornis*	Thrombin	IC_50_ ~ 300 nM	Unknown	Nakajima et al., 2006
Madanins	*Haemaphysalis longicornis*	Thrombin	*K*_D_ ~ 3–4 μM; *K*_i_ ~ 31–55 nM	Unknown	Iwanaga et al., 2003; Figueiredo et al., 2013
Variegin	*Amblyomma variegatum*	Thrombin	IC_50_ ~ 1 nM; *K*_i_ ~ 10 pM	Unknown	Kazimírová et al., 2002; Koh et al., 2007
NTI-2	*Hyalomma dromedarii*	Thrombin	*K*_i_ ~ 211 nM	3.8 mM–3.8 M	Ibrahim et al., 2001b
Americanin	*Amblyomma americanum*	Thrombin	*K*_i_ ~ 0.073 nM	100 nM–100 μM	Zhu et al., 1997a
Saliva	*Amblyomma americanum*	fXa; Thrombin	100% I ~ 0.3 SU	Unknown	Zhu et al., 1997b
Ornithodorin	*Ornithodoros moubata*	Thrombin	*K*_i_ ~ 1 pM	Unknown	van de Locht et al., 1996
Anticoagulant	*Ixodes holocyclus*	Clotting	IC_50_ ~ 0.25 SGU	Unknown	Anastopoulos et al., 1991
Ixin	*Ixodes ricinus*	Thrombin	IC_50_ ~ 0.6 SGU	Unknown	Hoffmann et al., 1991
Calcaratin	*Rhipicephalus calcaratus*	Thrombin	Unknown	Unknown	Motoyashiki et al., 2003
Haemathrin	*Haemaphysalis bispinosa*	Thrombin	IC_50_ ~ 40 μM	Unknown	Brahma et al., 2017
Microphilin	*Rhipicephalus microplus*	Thrombin	IC_50_ ~ 6–42 μM	17 μM–17 mM	Ciprandi et al., 2006
BmAP	*Rhipicephalus microplus*	Thrombin	IC_50_ ~ 100 nM–1 μM	333 nM–33 μM	Horn et al., 2000
NTI-1	*Hyalomma dromedarii*	Thrombin	*K*_i_ ~ 11.7 μM	650 μM–6.5 M	Ibrahim et al., 2001b
HLS2	*Haemaphysalis longicornis*	Thrombin	IC_50_ ~ μM	Unknown	Imamura et al., 2005
IRS-2	*Ixodes ricinus*	Cathepsin G; Chymase	IC_50_ ~ 4–11 nM	Unknown	Chmelař et al., 2011
AamS6	*Amblyomma americanum*	Undefined	All tests μM range; no parameters	Immunogenic	Chalaire et al., 2011; Mulenga et al., 2013
Penthalaris	*Ixodes scapularis*	Tissue factor pathway	IC_50_ ~ 100 pM	Unknown	Francischetti et al., 2004
Ixolaris	*Ixodes scapularis*	Tissue factor pathway fXa	IC_50_ ~ pM range	Unknown	Francischetti et al., 2002; Monteiro et al., 2005
Hd_fXaI	*Hyalomma dromedarii*	fXa	*K*_i_ ~ 134 nM	0.3 mM–300 mM	Ibrahim et al., 2001a
Anticoagulant	*Rhipicephalus appendiculatus*	fXa	450% clotting time increase for 2 SGU	100 nM–125 μM	Limo et al., 1991
TAP	*Ornithodoros moubata*	fXa	*K*_i_ ~ 0.6 nM	8 μM–8 mM	Waxman et al., 1990
fXaI	*Ornithodoros kalahariensis*	fXa	*K*_i_ ~ 0.8 nM	250 nM–250 μM	Gaspar et al., 1996
Salp14	*Ixodes scapularis*	fXa	IC_50_ ~ 150 nM	Immunogenic	Narasimhan et al., 2002
Amblyomin-X	*Amblyomma sculptum*	fXa	IC_50_ ~ 10 μM; *K*_i_ ~ 4 μM	Unknown	Batista et al., 2010; Branco et al., 2016
TIX-5	*Ixodes scapularis*	fV	IC_50_ ~ 3.2 μM; all tests μM range	Immunogenic	Schuijt et al., 2011, 2013
Rhipilin-1	*Rhipicephalus haemaphysaloides*	Elastase	SI ~ 6	Unknown	Cao et al., 2013
BmTI-A	*Rhipicephalus microplus*	Elastase; Kallikrein	*K*_i_ ~ 1.4 nM; *K*_i_ ~ 120 nM	Unknown	Tanaka et al., 1999
Iris	*Ixodes ricinus*	Elastase TNF-α	IC_50_ ~ μM IC_50_ ~ 50 nM	Unknown	Leboulle et al., 2002; Prevot et al., 2006, 2009
RsTIQ 2, 5, 7	*Rhipicephalus sanguineus*	Elastase; plasmin	*K*_i_ ~ 1–38 nM	Unknown	Sant’Anna Azzolini et al., 2003
Haemaphysalin	*Haemaphysalis longicornis*	fXIIa	IC_50_ ~ 50–100 nM; *K*_D_ ~ 3 nM	Unknown	Kato et al., 2005
AAS19	*Amblyomma americanum*	Plasmin; Thrombin	SI ~ 3–9	Unknown	Kim et al., 2015; Radulović and Mulenga, 2017
TdPI	*Rhipicephalus appendiculatus*	Tryptase	*K*_i_ < 1.5 nM	Unknown	Paesen et al., 2007
Tryptogalinin	*Ixodes scapularis*	Plasmin Tryptase	*K*_i_ ~ 5.83 nM *K*_i_ ~ 10 pM	Unknown	Valdés et al., 2013
Sialostatin L	*Ixodes scapularis*	Cathepsin L	IC_50_ ~ 5 nM; *K*_i_ ~ 95 pM	Detected in saliva	Kotsyfakis et al., 2006
Sialostatin L2	*Ixodes scapularis*	Cathepsin L	IC_50_ ~ 70 pM; *K*_i_ ~ 65 pM	Unknown	Kotsyfakis et al., 2007
Iristatin	*Ixodes ricinus*	Cathepsin L and C	IC_50_ ~ 500 nM–3 μM	Unknown	Kotál et al., 2019
OmC2	*Ornithodoros moubata*	Cathepsin L and S	IC_50_ ~ 150 pM; *K*_i_ ~ 65 pM	Detected in saliva	Grunclová et al., 2006; Salát et al., 2010
BrBmcys2b	*Rhipicephalus microplus*	Cathepsin B and L	*K*_i_ ~ 1–3 nM	Detected in saliva	Parizi et al., 2013
RHcyst-2	*Rhipicephalus haemaphysaloides*	Cathepsin S	IC_50_ ~ 100 pM	Detected in saliva	Wang et al., 2015
TCI	*Rhipicephalus bursa*	Carboxy-peptidase	*K*_i_ ~ 1–4 nM	150 nM–150 μM	Arolas et al., 2005
HlTCI	*Haemaphysalis longicornis*	Carboxy-peptidase	IC_50_ ~ 10 μM	Unknown	Gong et al., 2007
Evasin-1	*Rhipicephalus sanguineus*	CCL3; CCL8; CCL18	*K*_D_ ~ 160 pM; *K*_D_ ~ 810 pM; *K*_D_ ~ 3 nM	Detected in saliva	Frauenschuh et al., 2007
Evasin-3	*Rhipicephalus sanguineus*	CXCL1; CXCL8	*K*_D_ ~ 340 pM; *K*_D_ ~ 700 pM;	Detected in saliva	Déruaz et al., 2008
Evasin-4	*Rhipicephalus sanguineus*	CCL1-CCL27	*K*_D_ ~ 10–700 pM;	Detected in saliva	Déruaz et al., 2013
P1243 (AAM-02)	*Amblyomma americanum*	CCL1-CCL27	*K*_D_ ~ 1–100 nM	Unknown	Alenazi et al., 2018
P1156	*Ixodes ricinus*	CXCL1-8	*K*_D_ ~ 3–70 nM	Unknown	Alenazi et al., 2018
RPU-01, RPU-02, AAM-01, AAM-02, ACA-01, ACA-02, AMA-01, ATR-02, IRI-01	*Rhipicephalus pulchellus Amblyomma americanum Amblyomma cajennense Ixodes ricinus Ixodes holocyclus*	Eotaxin-1; Eotaxin-2; Eotaxin-3; MCP-1; MCP-2; MCP-3	*K*_D_ ~ 5–1,000 nM	Unknown	Hayward et al., 2018
P991, P985, P546, P974, P983, P1181, P1182, P1183, P1180, P467	*Amblyomma maculatum Amblyomma triste Amblyomma parvum Rhipicephalus pulchellus*	CCL2, CCL3, CCL4, CCL7, CCL8, CCL11, CCL13,CCL14, CCL16, CL17, CCL18,CCL19 CCL20,CCL21, CCL22, CL23 CCL24,CCL27, CCL28, CCL25	*K*_D_ ~ 1 pM–3 μM	Unknown	Singh et al., 2017
P672	*R. pulchellus*	CCL3, CCL8, CCL11, CCL13, CCL14, CCL16, CCL18, CCl23	*K*_D_ ~ 1–10 nM; IC50 ~ 2–6 nM	Unknown	Eaton et al., 2018
OMCI	*Ornithodoros moubata*	C5 complement	IC_50_ ~ 12–27 nM *K*_D_ ~ 18 nM *K*_D_ < 100 pM	29 μM–29 mM	Nunn et al., 2005; Hepburn et al., 2007; Macpherson et al., 2018
TSGP2	*Ornithodoros kalahariensis*	C5 complement	*K*_D_ ~ 26 nM	38 μM–38 mM	Mans et al., 2001; Mans and Ribeiro, 2008b
TSGP3	*Ornithodoros kalahariensis*	C5 complement	*K*_D_ ~ 14 nM	38 μM–38 μM	Mans et al., 2001; Mans and Ribeiro, 2008b
RaCI	*Rhipicephalus appendiculatus*	C5 complement	IC_50_-6–21 nM	Unknown	Jore et al., 2016
Isac; Salp9; Salp20	*Ixodes scapularis*	C3 complement	IC_50_ ~ 200 nM	Detected in saliva	Valenzuela et al., 2000; Soares et al., 2005; Tyson et al., 2007, 2008
IRAC; IxAC	*Ixodes ricinus*	C3 complement	IC_50_ ~ 10–50 nM	Unknown	Daix et al., 2007; Schroeder et al., 2007; Couvreur et al., 2008
HT1-12	*Ixodes holocyclus*	Presynapse	IC_50_ ~ 1–10 mM	Detected in saliva	Chand et al., 2016
Salp15	*Ixodes scapularis*	T cell proliferation IL2 production CD4	*K*_D_ ~ 47 nM	Detected in saliva	Anguita et al., 2002; Garg et al., 2006
Longistatin	*Haemaphysalis longicornis*	RAGE	*K*_D_ ~ 72 nM	Detected in saliva	Anisuzzaman et al., 2014
Adrenomedullin	*Ornithodoros moubata*	Vasodilation	IC_50_ ~ 7 nM	Unknown	Iwanaga et al., 2014
Ra-KLP	*Rhipicephalus appendiculatus*	maxiK channels	AC_50_ ~ 1 μM	Unknown	Paesen et al., 2009
Ixonnexin	*Ixodes scapularis*	Plasminogen-tPA	*K*_m_ ~ 70–200 nM *K*_D_ ~ 4–19 nM	Detected in saliva	Assumpção et al., 2018


*Indicated are relative affinity reported as the dissociation equilibrium constant (K_*D*_), inhibitory constant (K_*i*_), Michaelis constant (K_*m*_), stoichiometric inhibition ration (SI), or inhibitory concentration (IC_*50*_). Salivary gland unit (SGU) refers to a fraction of a salivary gland. Minimum and maximum concentrations were calculated based on yields per tick reported and a feeding site volume of 10 nl–10 μl. Were indicated that proteins were detected in saliva or salivary gland extract (SGE), this was done with either western blot, affinity pull-down or proteomics*.

## Tick Feeding and Modulation of Host Defenses

**FIGURE 5:**
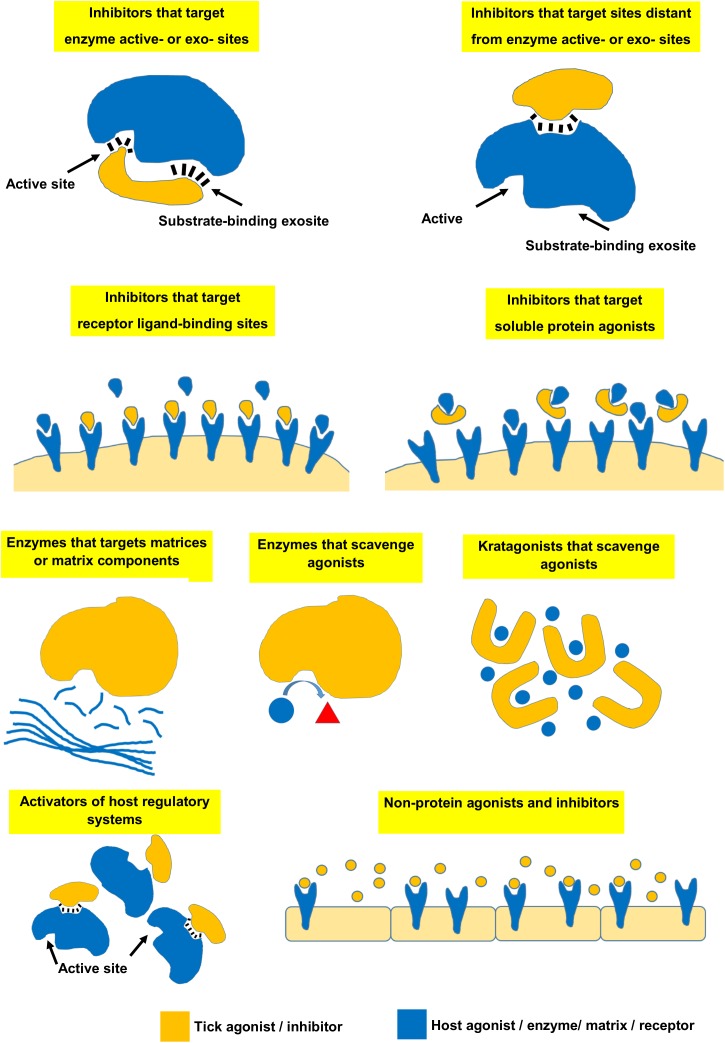
Various mechanisms used by ticks to modulate vertebrate host defenses.

Ticks need to modulate their vertebrate host’s defense mechanisms to obtain a successful blood-meal (Francischetti et al., 2009). In this regard, ticks secrete numerous proteins during feeding that function as inhibitors, enzymes, or kratagonists that use a variety of mechanisms to overcome host defenses (Mans, 2011; Mans et al., 2016) (Figure 5).

## Inhibitors That Target Enzyme Active Sites and/or Substrate-Binding Exosites

Various enzymes are involved in host hemostasis and many are part of enzyme cascades such as the blood clotting or complement cascade that enable efficient control as well as rapid response to injury or infection (Francischetti et al., 2009). Inhibitors for these cascades are found in all ticks. Inhibitors of blood-clotting enzymes generally target the active and exosites and inhibit primary enzymatic activity of thrombin, fXa, kallikrein, kallikrein-fXIIa–fXIa, fXa-TF-VIIa, plasmin, fV or carboxypeptidase B, thereby inhibiting formation or dissolution of the fibrin clot (Table 1 including references). Inhibitors may also target enzymes that may induce platelet aggregation such as cathepsin G, or enzymes involved in inflammation such as cathepsin B, C, H, L, S, and V, chymase, elastase, and tryptase (Table 1 and references therein). Targeting of enzyme active sites or substrate-binding exosites prevent binding of the substrate to the enzyme, thereby blocking its downstream effects. Most of these inhibitors have affinity measurements ranging from low pM to nM and for those whose concentrations could be estimated, would be present at relevant concentrations (Table 1).

With regard to host protein concentrations and affinities, prothrombin occurs at ~1.4 μM in plasma, although only sub μM quantities are generally converted to thrombin (Butenas and Mann, 2002). Fibrinogen and fibrin has a low affinity site for thrombin with *K*_D_ ~ 2–5 μM and a high affinity site with *K*_D_ ~ 100 nM, while occurring at a plasma concentration of ~7.6 μM. In addition, the *K*_D_ for the thrombin platelet receptor GP1bα is ~50–200 nM (Adams and Huntington, 2006). It is therefore not unexpected that inhibitors would have *K*_D_ values in the low nM or pM range, while also occurring at low nM to μM concentration ranges to allow efficient competition with fibrinogen. fX occur at a plasma concentration of ~170 nM, while fXa concentrations found in the clot range from 2 to 16 nM (Butenas and Mann, 2002). These concentrations also correlate with *K*_i_ values of tick inhibitors that are in the low nM range or below (Table 1). Other blood clotting factors have even lower concentrations ranging from low nM to pM concentrations in plasma (Butenas and Mann, 2002). Neutrophil derived proteases such as elastase or cathepsin G can reach concentrations of 100 nM at the site of release and is sufficient to induce platelet aggregation (Ferrer-Lopez et al., 1990). Again, the low nM IC_50_ or *K*_D_ values for the tick inhibitors correspond with the host target concentrations, supporting functional relevance at the tick feeding site (Table 1). Some clotting enzyme inhibitors have *K*_i_ or IC_50_ values in the μM ranges (Table 1). While estimates indicate that tick proteins may achieve such high concentrations at the feeding site, it remains crucial to confirm that they actually do.

## Inhibitors That Target Sites Distant From Enzyme Active Sites

Inhibitors may target enzymes at sites distant from the active site, thereby disrupting complex assembly and downstream activation and include inhibitors of C5 complement such as *Ornithodoros moubata* complement inhibitor (OMCI) and *Rhipicephalus appendiculatus* complement inhibitor (RaCI) (Nunn et al., 2005; Jore et al., 2016). OMCI binds to the C5d, CUB, and C345c domains of C5, while RaCI binds to the MG1, MG2, and C5d domains (Jore et al., 2016). In both cases, these domains and the inhibitor binding sites are distant from C5a or the C5 convertase binding sites suggesting that these inhibitors do not directly inhibit interaction of convertases with C5, but probably inhibit rearrangement of domains within C5 that is necessary for activation to occur (Jore et al., 2016). Other complement inhibitors from soft ticks include the OMCI homologs TSGP2 and TSGP3 from *O. kalahariensis* (Mans and Ribeiro, 2008b). These inhibitors have *K*_D_ values in the low nM range (Table 1). A recent study that used site-specific immobilization of OMCI and multicycle kinetics indicated that the *K*_D_ may even be in the low pM ranges indicating that assay design can affect estimations of affinity (Macpherson et al., 2018). These inhibitors also show quite high expected concentrations at the feeding site, which is mostly due to their functioning as kratagonists (section below). Complement C5 occur at a concentration of ~370 nM in plasma and its activator (C3/C5 convertase) show *K*_m_ values of 5–16 nM for the classical and alternative complement pathways (Rawal and Pangburn, 2001, 2003). Therefore, even at low nM concentrations and the high concentrations of inhibitor expected at the feeding site, the tick proteins should be functionally relevant. Hard ticks also possess complement inhibitors that belong to the Isac/IRAC family (Valenzuela et al., 2000). These inhibitors target the C3 convertase complex (C3bBbP) of the alternative pathway and dissociate this complex, preventing binding of the convertase to C3. IC_50_ values that range from 10 to 200 nM has been reported (Table 1). Of these inhibitors, Salp20 specifically target properdin of the C3 convertase complex and bind with a *K*_D_~0.6 nM, which is much lower than the *K*_D_ of properdin (>85 nM) for C3b (Tyson et al., 2008). As such, these inhibitors should be functionally relevant at the feeding site although affinities for all homologs have not yet been determined. The blood-clotting inhibitor ixolaris targets the heparin-binding exosite of fXa, thereby disrupting formation of the prothrombinase complex with an IC_50_ in the pM range (Francischetti et al., 2002; Monteiro et al., 2005). It would therefore be expected to be functionally relevant.

## Inhibitors That Target Receptor Ligand-Binding Sites

Inhibitors that target cell receptors thereby blocking binding of natural ligands and receptor activation includes fibrinogen receptor (GPIIbIIIa; α_IIb_β_3_) antagonists that inhibit platelet aggregation induced by any agonist (Karczewski et al., 1994; Wang et al., 1996; Mans et al., 2002b, 2008a; Tang et al., 2015). It has been shown that these inhibitors can also compete with bound fibrinogen to disaggregate aggregated platelets (Mans et al., 2002a). Binding to the fibrinogen receptor do not seem to result in outside-in signaling by these agonists. Inhibitors that target fibrinogen receptors need to be present at high concentrations, since platelets possess high numbers of the α_IIb_β_3_ integrin on their surface (~80,000 receptors/platelet) (Wagner et al., 1996). This could result in concentrations of active receptor of ~39 nM at the feeding site. The affinity constant (*K*_D_) for fibrinogen is ~1.4 μM, while fibrinogen occur in 10-fold excess (Frojmovic et al., 1994). It is therefore not unexpected that inhibitors would have *K*_D_ values in the nM range to bind to the receptor, while also occurring at μM concentrations to effectively compete with fibrinogen. Conversely, inhibitors with *K*_D_ or IC_50_ values above 1 μM may not be effective inhibitors.

Tick adhesion inhibitor (TAI) inhibits adhesion of platelets to collagen with an IC_50_ ~ 8 nM (Karczewski et al., 1995). Competitive inhibition (IC_50_ ~ 5 nM) of binding of the monoclonal antibody Gi9 to the platelet collagen receptor GPIa-IIa (α_2_β_1_) confirmed targeting of this receptor by TAI (Karczewski et al., 1995). The collagen receptor (GP1a/IIa) occur at low receptor numbers on platelets (800 receptors/platelet) resulting in a concentration of ~400 pM at the feeding site (Coller et al., 1989). The affinity of collagen for platelets is also ~35–90 nM (Jung and Moroi, 1998). It is therefore not surprising that the affinity for collagen receptor antagonists may be in the low nM range, with similar low nM concentrations at the feeding site.

Longistatin binds to the V domain of the receptor for advanced glycation end products (RAGE) with a *K*_D_ ~ 72 nM, thereby inhibiting ligand-induced inflammation in tissues (Anisuzzaman et al., 2014). It has been detected at the feeding site using host antibodies and would therefore presumably be present at nM concentrations (Anisuzzaman et al., 2010). The concentration for RAGE is in the low pM ranges (Bopp et al., 2008) and longistatin should therefore be able to saturate the receptor at nM concentrations.

Inhibitors that target receptors and upon binding induce antagonistic responses do exist. Salp15 binds to the CD4 coreceptor on T cells with a *K*_D_ ~ 47 nM and inhibits T-cell receptor ligation induced activation resulting in immunosuppression (Anguita et al., 2002; Garg et al., 2006). Salp15 also interact with DC-SIGN on dendritic cells (DC) to activate the serine∖threonine kinase Raf-1. This leads to modulation of Toll-like receptor induced DC activation (Hovius et al., 2008). However, in the latter case no affinity assessment was done, so it remains difficult to assess the biological relevance of this at the tick-feeding site. The CD4 coreceptor occurs at ~16–664 pM (Platt et al., 1998), and if Salp15 occur at concentrations equal or above its *K*_D_ for the CD4 coreceptor should saturate the receptor and will be biologically relevant. The neurotoxins from *Ixodes holocyclus* are presumed to target and inhibit presynaptic P/Q-type voltage gated calcium channels (Chand et al., 2016). Recombinant holocyclotoxins have IC_50_ values ranging from 5 to 12 μM, which seem to be orders of magnitude higher than the concentrations present in saliva (Chand et al., 2016), suggesting that a discrepancy still exist between the identified toxins and crude salivary composition (Pienaar et al., 2018).

## Inhibitors That Target Soluble Protein Agonists

A number of soluble host protein agonists exist that play a role in inflammation and immunity. These generally bind to receptors on leukocytes to activate cellular responses and cellular migration and are collectively known as cytokines or chemokines (Sokol and Luster, 2015). Leukocytes include eosinophils, mast cells, monocytes, neutrophils and natural killer cells that migrate along chemokine concentration gradients caused by release of chemokines from sites of infection or inflammation (Moore et al., 2018). A large number of chemokines exist that have specificities for different cell types. Chemokines are classified based on their conserved disulphide bond patterns. C chemokines possess a single disulphide bond and consist of two chemokines (XCL1 and XCL2). CC chemokines (β-chemokines) possess two disulphide bonds, with adjacent cysteines near the N-terminal and consist of 28 chemokines (CCL1–CCL28), described thus far. CXC chemokines (α-chemokines) possess two disulphide bonds with the adjacent cysteines near the N-terminal separated by a single amino acid and consist of 17 chemokines (CXCL1–CXCL17), described to date. A number of inhibitors from ticks that interact directly with these soluble agonists and prevent binding to their receptors have been described and are known as the evasins (Frauenschuh et al., 2007; Déruaz et al., 2008). It has been shown that evasins can bind to a wide array of cytokines or chemokines (Table 1). Their *K*_D_ values range from low pM to nM.

In the case of chemokines where leukocytes respond to concentration gradients for directional chemotaxis, the measured gradients suggest that these may range across low pM to several hundred nM (Moore et al., 2018). Naturally formed gradients depend on various factors that will influence the concentration gradient, notably, the type of cell secreting the chemokine, the amount secreted that depend on environmental cues, the presence of flow-induced shear stress and interaction of the chemokines with the extracellular matrix (Moore et al., 2018). Depending on the *K*_D_ of the evasins (low nM), chemotaxis may only be efficiently inhibited somewhere along the concentration gradient and not across the whole range, especially since competition of evasins for different chemokines might occur.

## Enzymes That Target Matrices or Matrix Components

Ticks feed from a feeding cavity where blood pools (Tatchell and Moorhouse, 1968). The feeding site needs to be remodeled to form this cavity (Wikel, 2017). Ticks may secrete a variety of enzymes that will enable such remodeling. This includes hyaluronidase that targets hyaluronic acid, a major component of the extracellular matrix (Neitz et al., 1978). Salivary transcriptomes also indicate that an abundant class of enzymes are the metalloproteases (Mans et al., 2016). While their role in feeding site remodeling has not been established beyond doubt, the general assumption is that these enzymes would play a role in remodeling (Mans, 2016; Wikel, 2017). Other metalloproteases that has been identified with a defined function include fibrin(ogen)ase activity that remove fibrinogen, both substrate for thrombin or platelet aggregation, or fibrin that forms the blood clot (Francischetti et al., 2003). Longistatin, a small EF-hand protein can hydrolyze α, β, and γ chains of fibrinogen, activates plasminogen to plasmin, degrade fibrin and dissolve fibrin clots (Anisuzzaman et al., 2011, 2012). Other enzymes that may target the fibrin clot without a direct interaction include plasminogen activators such as enolase that promote degradation of the fibrin clot via activation of the host enzyme plasminogen to plasmin (Díaz-Martín et al., 2013a; Xu X.L. et al., 2016). A serine protease that potentially activates protein C, a potent anticoagulant has also been identified in saliva of *Ixodes scapularis* (Pichu et al., 2014). For these “activating” enzymes the physiological effective concentrations in saliva may be low (pM–nM ranges) and detection of function in saliva (not salivary gland extract) may be enough to infer functional significance given their amplification/catalyzing nature.

## Enzymes That Perform Scavenging Functions

Some enzymes may perform scavenging functions by targeting bioactive molecules and catalyzing chemical reactions that inactivate or remove these molecules. This may prevent activation of receptors, or induce antagonistic responses in receptors by removal of activating ligand. In ticks, the enzyme apyrase (ATP-diphosphohydrolase; EC 3.6.2.5) hydrolyse ATP that function in inflammation (Faas et al., 2017), as well as ADP that induce platelet aggregation (Ribeiro et al., 1985, 1991; Mans et al., 1998a, 2008a). Apyrase was able to disaggregate platelets aggregated by ADP and caused platelet shape change from an activated spherical back to discoid form (Mans et al., 1998b, 2000), suggesting that bound ADP could be scavenged from its platelet receptor. Apyrase activity has been found in saliva or salivary glands of most ticks studied and have been assigned to the 5′-nucleotidase family (Stutzer et al., 2009). Family members have been found in all tick transcriptomes studied to date (Mans et al., 2016). Kinetic parameters from purified apyrase indicated a *K*_m_ ~ 1 mM for ATP and ADP and high turnover number (10^6^ s^-1^) and *K*_cat_/*K*_m_ ratio (10^9^ M^-1^s^-1^) (Mans et al., 1998b). These numbers indicate a highly efficient enzyme that would rapidly hydrolyze high local concentrations of ADP or ATP. Given the relatively high concentration of apyrase found in tick salivary glands (Mans et al., 1998b), it would probably also be functionally relevant. Another enzyme found in ticks that perform scavenging functions is a metallo dipeptidyl carboxypeptidase responsible for salivary kininase activity and breakdown of anaphylatoxin and bradykinin, involved in inflammation, pain and vasoconstriction (Ribeiro and Spielman, 1986; Ribeiro and Mather, 1998; Bastiani et al., 2002). In all cases of “scavenging” enzymes, the enzymes need to be able to rapidly remove host-derived agonist to levels below their functional ranges.

## Kratagonists That Perform Scavenging Functions

Kratagonists are related to “scavenging” enzymatic functions, by scavenging or mopping up of bioactive molecules, but without chemically changing their structures. Kratagonists may have similar functional activity as “scavenging” enzymes, such as preventing activation of receptors by ligands, or competitive removal of ligand from receptors causing an antagonistic response (Ribeiro and Arcà, 2009; Andersen and Ribeiro, 2017). The term kratagonist was recently coined to describe the abundant proteins found in saliva of most hematophagous organisms that function in a scavenging capacity (Ribeiro and Arcà, 2009). The name derives from the Greek “to arrest or to seize” and was appropriately proposed independently by the Greek compatriots Babis Savakis and Michalis Kotsyfakis (Ribeiro and Arcà, 2009). Recently the etymology of the name was redefined to indicate “hold” or “grab/capture” and “agonist” (Andersen and Ribeiro, 2017; Arcà and Ribeiro, 2018). In ticks a large number of kratagonists have been described, that all belong to the lipocalin family. Lipocalin structure is composed of an eight stranded anti-parallel β-barrel closed off at one end by an N-terminal 3_10_-helix, with a C-terminal α-helix anchored to the side of the barrel by disulphide bonds. This gives lipocalins the distinct appearance of a cup with an open end, where ligands can access the cup and bind in the cavity inside the barrel. Specificity is conferred by residues inside the barrel, as well as four loops that allow access to the barrel (Flower, 1996). The original name of lipocalin was assigned to “extracellular proteins capable of enclosing lipophiles within their structure to minimize solvent contact” (Pervaiz and Brew, 1987). Subsequently, lipocalins were defined based on conserved sequence or structural motifs (Flower, 1996). Scavenging functions performed by tick lipocalins include scavenging of histamine and serotonin (Paesen et al., 1999; Sangamnatdej et al., 2002; Mans et al., 2008b; Neelakanta et al., 2018), leukotriene B4 (Beaufays et al., 2008; Mans and Ribeiro, 2008b; Roversi et al., 2013), leukotriene C4 (Mans and Ribeiro, 2008a), thromboxane A2 (Mans and Ribeiro, 2008b), and cholesterol (Preston et al., 2013; Roversi et al., 2017).

In the case of small chemical agonists that are scavenged by either enzymes or kratagonists, the concentrations at which they activate their respective receptors are important. Platelets secrete ADP and ATP at 3–7 μM concentrations, which are also the concentration necessary for primary and secondary aggregation (Packham and Rand, 2011). Apyrase from ticks has been shown to effectively inhibit platelet aggregation at these activator concentrations, at enzyme concentrations well below the expected secretory levels (Mans et al., 1998b). Basophils and mast cells may secrete histamine to attain local concentrations of 20 μM in the skin that can lead to inflammatory responses when histamine binds to its receptors with *K*_D_ ~ 10 nM–30 μM (Petersen, 1997; MacGlashan, 2003). Serotonin is secreted by platelets at local concentrations of ~5 μM where it can cause vasoconstriction and platelet aggregation by binding to various serotonin receptors with *K*_i_ ~ 10 nM–1 μM (Watts et al., 2012). The very high concentrations of biogenic amine binding kratagonists at the feeding site (μM–mM) and their low affinities (low nM) indicate that they will be biologically relevant at the feeding site (Table 1).

Leukotriene B_4_ secreted by neutrophils may reach high concentrations at the site of neutrophil release (~950 nM) (Lewis et al., 1982). The affinity of LTB_4_ for its neutrophil receptor BLT1 ranges from 0.1 to 2 nM (Yokomizo, 2015). Again, the low affinities observed for the LTB_4_ scavengers and their high concentrations (μM) at the feeding sites would make them relevant competitors at the feeding site.

Cysteinyl leukotrienes can only be detected in plasma during inflammatory reactions such as asthma attacks and then occur at concentrations of ~100–765 pM (Sasagawa et al., 1994). It has been shown to cause vasoconstriction and vasopermeability at a concentration of ~100 nM, and bind with affinities from 5 to 35 nM to its receptors (Drazen et al., 1980; Krilis et al., 1983; Ghiglieri-Bertez et al., 1986; Prié et al., 1995). Scavengers of LTC_4_ and LTD_4_ have *K*_D_ values below 2 nM and also occur at μM concentrations (Table 1). It is therefore also expected that they would be physiologically relevant during feeding.

Thromboxane A_2_ is released from platelets at concentrations of 11–35 nM, which is capable of inducing platelet aggregation and bind to platelet receptors with *K*_D_ ~ 4 nM (Hamberg et al., 1975; Dorn and DeJesus, 1991). The high concentrations of the TXA_2_ kratagonists at the feeding site (μM–mM) and their low affinities (low nM) suggest that they would be able to neutralize TXA_2_ binding to its receptor during feeding (Table 1).

## Activators of Host Regulatory Systems

The majority of tick host modulatory mechanisms described so far comprise inhibitors. However, activators may also play important roles since these may target the natural regulatory feedback systems of host hemostasis. Plasminogen activators that result in degradation of the fibrin clot have already been discussed. Recently, a small peptide named ixonnexin that belongs to the basic tail family was described that act by promoting interaction of plasminogen and tissue plasminogen activator (tPA) by forming a enzymatically productive ternary complex that forms plasmin to promote fibrinolysis (Assumpção et al., 2018). Ixonnexin interacts with both plasminogen and tPA with similar *K*_D_ values (4–20 nM). Such an activator would need to be present at similar or higher concentrations than the target enzymes to be functionally relevant. In the case of ixonnexin, plasminogen and tPA occur at plasma concentrations of ~2 μM and 100 pM, respectively (MacGregor and Prowse, 1983; Assumpção et al., 2018). While no exact concentration has been established for ixonnexin, it has been estimated to occur at high concentrations in saliva (Assumpção et al., 2018). It has also been suggested that all basic tail proteins may perform this function, including the fXa inhibitor Salp14, since this family in which the C-terminal is rich in basic amino acids such as lysine and arginine mimics the C-terminal lysine present in fibrin that serves as recognition site for plasminogen and tPA lysine binding sites (Assumpção et al., 2018).

The adrenomedullins are a special case of tick-derived inhibitors that bind to host calcitonin-receptor-like receptor and receptor activity-modifying protein receptor complexes to cause vasodilation (Iwanaga et al., 2014). These inhibitors have not converged to mimic host adrenomedullin, but have been acquired via horizontal gene transfer from a mammalian host (Iwanaga et al., 2014). At systemic concentrations of ~7 nM it reduced blood pressure by almost 50% (Table 1). It may therefore be assumed that secretion of such low quantities during feeding may result in local vasodilatory effects. However, it’s presence in saliva at functional concentrations still needs to be confirmed.

An activator of MaxiK channels have been described in *R. appendiculatus*, where it presumably play a role in regulation of blood vessel tonus and blood flow (Paesen et al., 2009). It invoked a half maximal response in MaxiK channels at 1 μM. Its functionality at the feeding site remains to be resolved.

## Non-protein Agonists and Inhibitors

Ticks may also secrete non-protein agonists that could affect the host’s defense mechanisms. As such, ixodid ticks secrete prostaglandin E2 (PGE2), a potent vasodilator, at ~40–500 ng/ml saliva (Ribeiro et al., 1985, 1992; Inokuma et al., 1994). Concentrations of PGE2 may range from 79 nM to 994 μM in adults at the feeding site, which would be pharmacologically active, since the *K*_D_ of the PGE2 receptor is ~0.7 nM (Davis and Sharif, 2000). Another non-protein mechanism for modulating host inflammatory and pain sensing responses was recently reported that involved secretion of saliva-specific microRNAs that was detected in salivary exosomes using next-generation sequencing (Hackenberg et al., 2017; Hackenberg and Kotsyfakis, 2018). The efficiency of exosomal miRNA will depend on the concentrations of exosomes in saliva, the specificity of the exosomes for specific cell types and the concentration of miRNA inside the exosomes (Hu et al., 2012). As yet, the functional relevance of saliva-derived exosomal miRNA still needs to be confirmed, with their kinetics of inhibition resolved, since the question remains whether a single exosome would only target a single lymphocyte or epithelial cell, in which case the effectiveness of inhibition would be determined by the number of cells that can be neutralized at the feeding site. It would, however, add another complex repertoire to the ticks expanding modulatory mechanisms.

## Strategies to Circumvent the Affinity/Equilibrium Barrier

**FIGURE 6:**
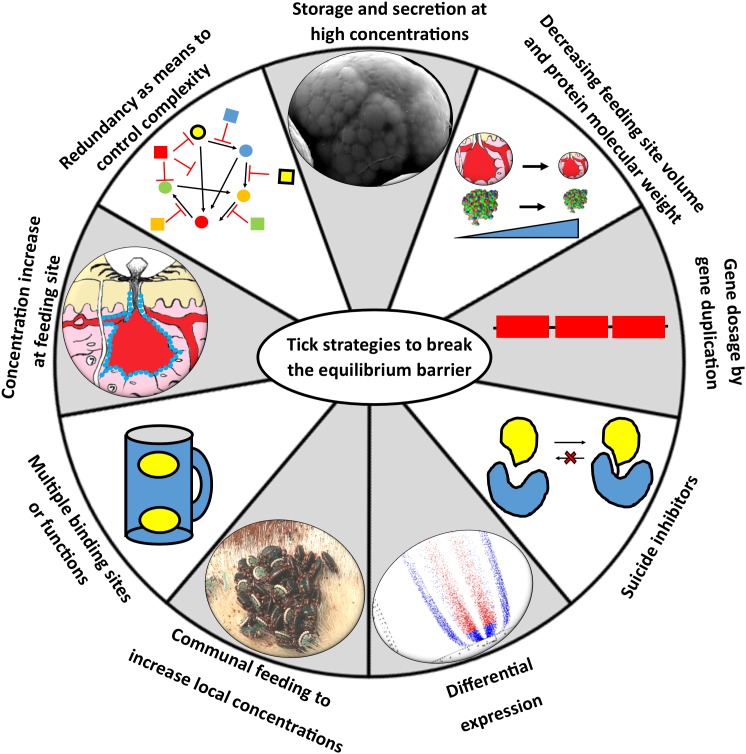
Strategies to circumvent the equilibrium barrier. Ticks can use various strategies to increase local concentrations at the feeding site to satisfy equilibrium binding conditions.

It is evident that the majority of tick proteins thus far characterized would have physiological functionality at the feeding site (Figure 3 and Table 1). However, expression and secretion of high concentrations of secretory proteins via the salivary secretory granules may be restricted to very few proteins given physical constraints of granule packaging (Mans and Neitz, 2004b). The dynamic nature of the feeding site may also make the actual concentrations of tick proteins present at any given moment much more haphazard than expected. The concentrations at the feeding site may very well be 100–10,000-fold lower than estimated, in which case concentrations may drop to pM–nM for most proteins, which may be below the *K*_D_ values for many proteins. To address this, ticks may employ various alternative strategies that allow adequate expression and optimal use of secreted proteins at the feeding site that allow ticks to circumvent or eliminate the problems posed by an equilibrium system (Figure 6). The next section discusses such strategies in more detail.

### Redundancy as Means to Control Complexity and Chaos

The host’s defense systems such as the clotting, platelet aggregation, complement and inflammatory cascades are part of one big redundant integrated feedback system that allows rapid response as well as control of the system (Delvaeye and Conway, 2009; Deppermann and Kubes, 2016; Wiegner et al., 2016). It has been argued that this redundancy is mimicked in the complexity of functions observed in the salivary glands of blood-feeding arthropods (Ribeiro, 1995). Conversely, it has been argued that the complexity observed in the functional salivary repertoire of ticks is due to a highly optimized system of defense shaped by evolution (Nuttall, 2019). However, redundancy may serve the purpose of dealing with a highly dynamic and chaotic system, as may be seen at the feeding site, which is in constant flux with ever changing concentrations of target and inhibitor molecules. Targeting of many different host proteins at once, even if not at optimal concentrations, may disrupt a redundant system enough to allow successful feeding. In this environment no protein needs to be 100% effective, but only good enough to get the job done. This may allow non-optimal proteins to function at the feeding site, or be maintained while evolving more optimized functions as postulated in the playground hypothesis of neutral evolution (Mans et al., 2017). The question then becomes a matter of how much inhibition of the host’s defense systems would be necessary to ensure successful feeding. In this regard, three observations could be made: firstly, that inefficient inhibition was surely a given during the early stages of adaptation to a blood-feeding lifestyle since protein functions were still optimized by natural selection. Secondly, since ticks had to switch hosts throughout their evolution, we have no evidence that ticks are not still adapting to new hosts, and functions that seem to be only partially effective may indeed still be optimized in future through natural selection. Thirdly, given the relatively high concentrations that may be obtained at the feeding site, even if proteins may only be partially effective, may allow for selection of these non-optimal functions to improve efficiency, again supporting a neutral evolution of function hypothesis (Mans et al., 2017). These arguments should, however, not serve as a *carte blanche* to support every claim of functional significance in blood-feeding, since the contribution of salivary derived proteins to species fitness has not been elucidated yet or even proven beyond doubt.

### Fast Feeding, Storage and Secretion of a Large Bolus of Salivary Proteins

**FIGURE 7:**
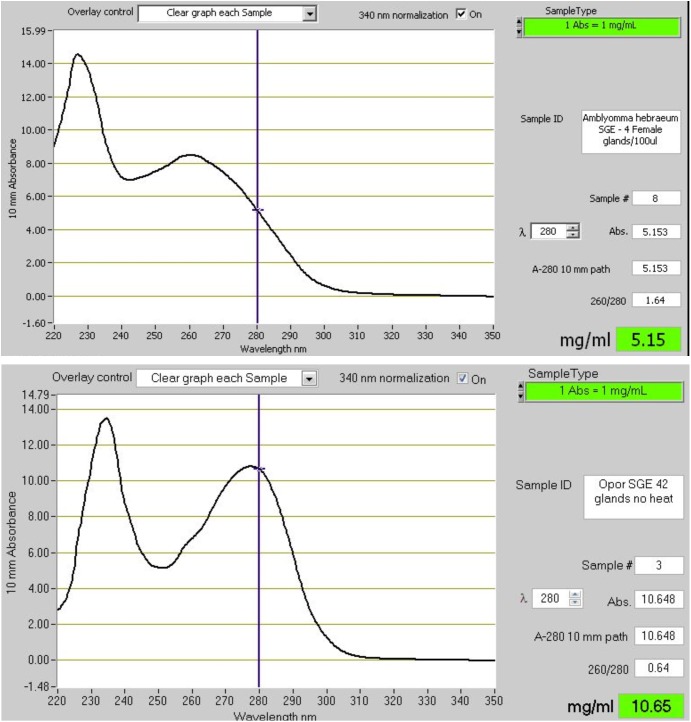
The absorbance spectra of salivary gland extracts (SGE) from hard and soft ticks. The top graph shows the spectrum of SGE of 4 glands from *Amblyomma hebraeum* suspended in 100 μl water. Based on the 280 nm absorbance a single gland would have a soluble protein concentration of ~128 μg. Bradford determination indicated a concentration closer to ~60 μg/gland. The bottom graph shows the spectrum of SGE of 42 glands from *Ornithodoros phacochoerus* suspended in 500 μl water. Based on the 280 nm absorbance a single gland would have a soluble protein concentration of ~127 μg. Bradford determination indicated a concentration of ~125 μg/gland. These absorbance spectra are representative of hard and soft tick SGE in general.

Soft ticks feed within minutes to hours to repletion, drop off, digest the blood meal slowly over the course of weeks to months, lay a small egg batch and can then feed several times more using the same pattern (Mans and Neitz, 2004a). During fast feeding most salivary proteins may be secreted in the course of 10–30 min and will be replenished within several days after feeding. Secretory proteins are stored in large granules up to 10 μM in diameter that effectively fill the salivary gland cells to their maximum extent (Mans and Neitz, 2004b; Mans et al., 2004b). The protein profiles of soft tick SGE attest to this, since protein spectra rarely show the presence of genomic DNA, in contrast to SGE from hard ticks that predominantly show genomic DNA/RNA, while the protein peak and concentration is obscured (Figure 7). This strategy from soft ticks allows concentrations of proteins that can overcome relatively high equilibrium dissociation constants by sheer concentration effects alone. Hard ticks utilize this strategy to some extent, since different salivary gland cells are filled with secretory granules over the course of the feeding period that can last several days to weeks (Binnington and Kemp, 1980). However, the amount of protein found in crude salivary gland extract rarely exceeds the concentrations of genomic DNA and is generally less than what would be observed for soft ticks (Figure 7), and may range from 5 to 60 μg total soluble protein over the course of feeding for a small tick such as *R. appendiculatus* (Wang and Nuttall, 1994). Similarly, concentrations in pilocarpine-induced salivary secretions in hard ticks are generally lower than soft ticks, with soft ticks attaining ~20–40 mg/ml and hard ticks ranging from 700 to 60 μg/ml over the course of feeding (Howell et al., 1975; Ribeiro, 1987; Ribeiro et al., 1991; Dharampaul et al., 1993; Chand et al., 2016).

### Communal Feeding to Increase Local Concentrations at the Feeding Site

Hard ticks feed for several days to weeks and mating on the host (or off the host for some *Ixodes* species) during the slow pre-feeding phase is required before the rapid engorgement phase can occur (Kiszewski et al., 2001). To allow mate finding, males secrete attraction-aggregation-attachment pheromones that result in ticks clustering and co-feeding at the same feeding site (Sonenshine, 2004). In a similar manner, specific tick species generally have preference sites of attachment on the host, presumably due to host environmental cues. While males do not engorge or take a significant blood meal, they do attach and secrete salivary components into the communal feeding site that apparently assist females in blood meal acquisition (Wang et al., 1998, 2001a). Creation of a communal locality where all ticks contribute to the localized but systemic feeding site may result in combined concentrations that overcome affinity restricted barriers.

### Gene Dosage and Cumulative Contributions From Multigene Families

Proteins may be maintained as gene duplicates to allow high level expression from each family member (Mans et al., 2017). While each member may by itself completely inhibit host functions, even partial inhibition by each gene duplicate can result in complete inhibition. This is a relatively simple example of the gene dosage effect that has been observed for fibrinogen receptor antagonists (Mans et al., 2003a, 2008a) and LTB_4_ scavengers (Mans et al., 2003b; Mans and Ribeiro, 2008b). It has been proposed that multigene families express different antigenic variable family members at low concentration levels to escape the immune system, the varying epitope hypothesis (Couvreur et al., 2008; Chmelař et al., 2016b). In this scenario, the low-level expressed proteins cumulatively target the same receptor to attain a combined concentration that would allow receptor saturation and inhibition. This may be possible, even if expressed at concentrations well below the equilibrium dissociation constant, since the inhibitory effects may be summed if the inhibitors act in a mutually exclusive manner (1:1 receptor-ligand binding with no synergistic effects) (Chou and Talalay, 1977). A cumulative effect was recently shown for the holocyclotoxins from the paralysis tick, *Ixodes holocyclus*, where at least 19 holocyclotoxin genes were expressed at low levels that showed a cumulative paralysis effect when combined (Rodriguez-Valle et al., 2017). It should be noted that while immune evasion was proposed as reason for the multiplicity of gene family members, low level expression of multiple genes to attain a cumulative threshold concentration that allows effective inhibition may also be possible and may fit with the overall neutral evolution of tick salivary gland proteins previously proposed (Mans et al., 2017). Low-level expression may in this scenario also be a function of the constitutive differential expression observed in hard ticks, where the extreme high expression levels observed in soft ticks due to accumulation in granules may not be attainable. Even so, it remains to be quantitatively proven that cumulative contributions to attain a threshold concentration occur at the feeding site.

### Increasing Concentration at the Feeding Site

The vertebrate host uses localized cues and responses to maintain and regulate haemostasis. As such, wounds or breakdown in system integrity are detected by exposure of localized collagen or other extracellular matrix components, activation of platelets and exposure of procoagulant platelet surfaces, initiation of blood-clotting and the complement cascade on activated platelet surfaces (Delvaeye and Conway, 2009; Deppermann and Kubes, 2016; Wiegner et al., 2016). In a similar manner, targeting of tick-derived bioactive molecules to activated surfaces, whether tick or host surfaces can increase local concentrations and prevent loss or dilution of components via blood meal ingestion or systemic diffusion. Salivary proteins have been found in tick cement where they presumably function as inhibitors of host defenses, or to prevent recognition of cement as a foreign or activation surface for host defenses (Bullard et al., 2016). Proteins such as apyrase have asymmetric charged surfaces (Stutzer et al., 2009), suggesting that these would be attracted to activated negatively charged platelet membranes. Other tick proteins has been shown to interact with membranes or have higher inhibitory activity in the presence of membranes, suggesting that these proteins may be concentrated on activated membrane surfaces at the feeding site (Ehebauer et al., 2002; Schuijt et al., 2013). Some Kunitz-domain inhibitors such as ixolaris have long carboxy-termini rich in serine and threonine residues that can be the site of glycosylation (Francischetti et al., 2002). These would create mucin tails that would be sticky and allow concentration on the walls of the feeding cavity preventing removal and increasing concentration (Francischetti et al., 2009). This may be a general strategy for glycosylated tick proteins (Uhlír et al., 1994).

### Increasing Effective Concentration Using Multiple Binding Sites or Multiple Functions

In the case of some tick lipocalins their effective concentration is increased by possessing two independent binding sites for histamine or one for histamine and one for serotonin (Paesen et al., 2000; Sangamnatdej et al., 2002; Mans et al., 2008b). This increase their capacity for scavenging twofold. Another variation on this may be proteins that can potentially target many independent ligands or targets and may therefore have multiple functions. While this certainly expands the potential of proteins to work within a complex redundant environment, the most effective of these would be proteins that have independent function and mechanisms. For example, targeting of complement C5 and LTB_4_ allows OMCI and its homologs to inhibit both complement and LTB_4_ mediated inflammation at the same time (Nunn et al., 2005; Mans and Ribeiro, 2008b; Roversi et al., 2013). In this case, both functions target the two key non-redundant mediators of neutrophil recruitment during inflammation that seems to be intricately linked (Sadik et al., 2018). This is a remarkable convergence of function in a single protein (Mans et al., 2017). On the other hand, if the functional mechanisms are not independent, i.e., use the same site for ligand binding or the same protein surface to target different proteins, competition between targets will effectively lower the concentration of available inhibitor, thereby impacting its functionality. This may be seen for moubatin and its homologs that bind both LTB_4_ and TXA_2_ within the same lipocalin cavity at similar affinities (Mans and Ribeiro, 2008b). If both LTB_4_ and TXA_2_ are present at similar concentrations, the effective scavenging capability may be halved. One way to compensate for this may be multiple proteins or very high concentrations at the feeding site as observed for moubatin, TSGP2 and TSGP3. The same problem would face many of the enzyme inhibitors that can inhibit two or more enzymes from a specific class such as the cystatins, where the presence of many different host enzymes at the feeding site would lead to competition with the tick inhibitors. Similarly, the evasins can bind to many chemokines with similar affinities that may lead to competition of evasins for different chemokines, thereby reducing their effectivity.

### Suicide Inhibitors

Inhibitors that bind in a 1:1 manner to their target enzymes, and then serve as substrate, resulting in the formation of a covalent enzyme-inhibitor complex, circumvent the equilibrium dissociation problem completely. Their initial affinities need only to be high enough for the enzyme to catalyze its reaction and form the covalent complex to result in permanent inhibition. In ticks, such inhibitors are found in the serpins that target various enzymes of the clotting cascade (Chmelař et al., 2017). Serpins form covalent complexes with their respective serine proteases after cleavage (Whisstock et al., 2010).

### Differential Expression

Ixodid ticks express different proteins at different periods during feeding (McSwain et al., 1982; Paesen et al., 1999; Wang et al., 2001b; Kim et al., 2017; Perner et al., 2018). Possible reasons proposed for this include antigenic variation to escape the immune system (Chmelař et al., 2016b), or responses to changes at the feeding site such as wound healing (Francischetti et al., 2005). Differential expression may also occur as salivary gland morphology changes during the course of feeding and different cell types play different roles in the feeding process (Binnington and Kemp, 1980). As such, differential expression may allow increased concentration spikes of proteins in specific feeding windows.

### Minimizing Feeding Site Volume

Comparison of adult, nymphal, and larval concentrations at the feeding site indicates that the relative concentrations remain constant between the various life stages even though salivary gland concentrations may differ several fold (Figure 3). This is mostly due to the differences observed in feeding cavity size that scale relative to the life stage and tick size. If this observation holds for all tick species it would imply that smaller ticks compensate by creating smaller feeding cavities. Feeding cavity size may then be related to protein concentration secreted during feeding.

### Smaller Proteins Allow Higher Molar Concentrations

The majority of secretory salivary proteins in ticks have low molecular masses below 25 kDa. This include the majority of highly abundant protein families such as the basic tail secretory, Kunitz-BPTI and lipocalin families (Mans et al., 2008c, 2016; Mans, 2011). Lower molecular masses allow higher relative molar concentrations at the feeding site, which could result in an up to a 10-fold difference between a 5 and 50 kDa protein (Figure 3).

## Functional Relevance and Mechanism

Understanding the mechanism of action (how a given protein works) is important in accessing functional relevance. As example, the case of moubatin may be considered. Originally moubatin was identified as a specific inhibitor of collagen-induced platelet aggregation with an IC_50_ ~ 50 nM, that did not affect ADP, arachidonic acid, thrombin, ristocetin, or calcium ionophore A23187 induced platelet aggregation (Waxman and Connolly, 1993). Subsequently, recombinant moubatin was shown to inhibit collagen-induced platelet aggregation with IC_50_ ~ 100 nM but did not inhibit adhesion to collagen (Keller et al., 1993). At high concentrations (5.8 μM) of moubatin and low concentrations of ADP (2 μM) 40% inhibition of ADP-induced platelet aggregation was observed, suggesting that the cyclooxygenase pathway may be targeted. It was also shown that moubatin at these high concentrations could inhibit the TXA_2_ mimetic U46619 and competed with the TXA_2_ receptor antagonist SQ29548 for binding to platelet membranes with IC_50_ ~ 10 μM (Keller et al., 1993). The leech inhibitor LAPP did not compete with SQ29548 binding to platelets, indicating that different receptors were targeted. Moubatin also did not inhibit binding of the monoclonal antibody Gi9 that inhibited adhesion to collagen and interacts with integrin α_2_β_1_, the proposed receptor for adhesion to collagen. At the time, moubatin did not share sequence homology with any inhibitor of collagen-induced platelet aggregation or with collagen, and did not contain the RGD motif important in integrin recognition. From the complex data above it was suggested that moubatin might be a TXA_2_ receptor antagonist (Keller et al., 1993). Subsequently, it was shown that moubatin’s mechanism of collagen-induced platelet aggregation is exclusively via scavenging of TXA_2_ with *K*_D_ ~ 20 nM, by gain and loss of function mutations in TSGP2 and TSGP3, respectively, two closely related homologs (Mans and Ribeiro, 2008b). In retrospect, it may be considered that the only inhibitory paradigms at the time were interaction with either collagen or specific platelet receptors and that the kratagonist paradigm as formulated recently did not exist. Interpreting the moubatin results from the previous paradigms may have been logical, even if the high IC_50_ observed for SQ29548 should have raised flags. It may now be suggested that moubatin was scavenging SQ29548 (a TXA_2_ mimetic), albeit with low affinity and did not compete for the receptor. Several lines of evidence converged on moubatin as scavenger of TXA_2_: the evidence that moubatin belonged to the lipocalin family (Paesen et al., 1999; Mans et al., 2003b); the fact that lipocalins are highly abundant in salivary glands and that abundant proteins generally act as scavengers, i.e., the kratagonist paradigm (Mans et al., 2001, 2003b; Mans and Neitz, 2004a,b; Calvo et al., 2006); the inhibitory effect of moubatin on the TXA_2_ mimetic U46619 (Keller et al., 1993), and the observation that a closely related protein, OMCI bound ricinoleic acid, suggesting that moubatin may bind prostaglandins and thromboxanes (Roversi et al., 2007). The gain of function mutation in TSGP2 of R85G and a similar complete loss of function for TSGP3 with the mutation G85R, allowed unambiguous confirmation of functional relevance as TXA_2_ scavengers (Mans and Ribeiro, 2008b). It not only highlighted the reductionist paradigm in elucidation of function, but also showed how elucidation of mechanism may inform on which function is considered relevant. As such, moubatin is an inhibitor of collagen-induced platelet aggregation, but perform this function by scavenging the secondary agonist TXA_2_. Its mechanism is primarily as kratagonist and not as receptor or ligand neutralizing inhibitor. Once mechanism is clarified the parameters necessary to assess functional significance can be better defined. In this case, that any homolog has to bind TXA_2_ in the low nM range, be present at high concentrations and possess the R85G substitution to be functionally relevant.

## Systems Biology, Bioinformatics and Functional Relevance

While the majority of functions found in ticks may be assigned functional significance (Table 1), enough reports in the literature indicate that caution should be exercised when evaluating functional relevance. This is compounded by advances in technology that allow systems approaches to the analysis of salivary gland protein dynamics and bioinformatics that allows functional analysis *in silico*. As such, recent advances in technology, both in next-generation transcriptome sequencing and proteomics, has allowed an unprecedented view of salivary gland dynamics from a systems perspective (Schwarz et al., 2014; Chmelař et al., 2016a; Mans et al., 2016; Kim et al., 2017). This has indicated how ticks differentially up- or down-regulate proteins during various time intervals in feeding, which suggest that ticks actively respond to the feeding environment, reflecting a fine tuned adaptation to the hosts defense mechanisms. While the systems paradigm clearly show how dynamic expression may be in ticks, inferences regarding function rests on inference by homology or annotation. For example, a recent excellent proteomic study followed the expression profile of *I. scapularis* sampled every 24 h until detachment and indicated differential expression for a large number of lipocalins annotated as histamine-binding proteins (Kim et al., 2017). The discussion focused on the functionality of lipocalins as histamine scavengers and how the data would support tick responses to host immunity and feeding. Interestingly, a functional study into biogenic amine binding lipocalins that specifically targeted *I. scapularis* lipocalins with biogenic amine binding motifs, failed to find any histamine-binding lipocalins, but only identified serotonin-binding lipocalins with a single binding site (Mans et al., 2008b). While this does not exclude the possibility that histamine-binding lipocalins exist in *Ixodes* ticks, the results thus far do not support it. Similarly, bioinformatic analysis predicted high affinity binding of histamine and serotonin in lipocalins from *Ixodes ricinus*, for which none of the critical residues involved in biogenic amine binding was conserved (Valdés et al., 2016). Molecular docking also recently predicted nM affinities for cystatins from *Ixodes persulcatus* without experimental verification of affinities or target enzymes (Rangel et al., 2017). While such bioinformatic and systems approaches can certainly direct research to proteins of interest, the data should be used with caution to infer functional relevance, especially if the possibility exists that some of these proteins may be only transiently expressed or at concentrations too low for functionality. For systems biology to come of age, we need accurate dissection of the feeding site in real-time to quantify fluxes in protein concentration while performing quality control with validated functions.

## Is Functional Relevance Relevant?

It may be considered whether it really matters whether a measured function is relevant during feeding, given the emerging recognition that all proteins may be moonlighting to some extent. From this perspective, any function present in tick saliva should be relevant at some level and our goal for the next few decades would be to assign functions to salivary proteins, whether relevant or not. A more comprehensive understanding would later emerge once we have gathered enough data to truly assess relevance. This position is appealing since it buys some time for dubious functions. It is, however, also a philosophical “everything goes” viewpoint (Feyerabend, 1975), that makes distinguishing important from trivial function very difficult. The same issue has been raised with regard to whether all ticks are venomous, or whether only some ticks secrete toxins that may cause the various well recognized forms of tick paralysis and toxicoses (Pienaar et al., 2018). By treating all ticks as venomous, the meaning of toxicity is obscured. Similarly, by treating all functions in saliva as relevant at the feeding site, even if their functional parameters suggest that they would not be relevant, may obscure those central in the feeding process from peripheral functions.

## Functional Relevance From a Practical Perspective

The use of tick salivary proteins as therapeutic agents within a clinical or pharmaceutical setting remains an important and promising goal (Mans, 2005). From this perspective any function determined for a protein need not be functionally relevant at the tick feeding site, as long as the specific parameters for use has been determined that would allow it to function under clinical or therapeutic controlled conditions. For example, the half-life of OMCI could be improved >50-fold by PASylation, making it more relevant for clinical use (Kuhn et al., 2016). In a similar vein, anti-tick vaccines may be developed against proteins with unknown functions or even irrelevant function, as long as the vaccine shows efficacy, as for example for hidden antigens (Nuttall et al., 2006). On the other hand, development of vaccines against exposed antigens may work better if antigens with real functional significance at the feeding site can be defined, their mechanism of action elucidated and this information used to rationally design target strategies that would neutralize function at the feeding site effectively.

## Conclusion

Functional relevance is determined by the concentration of tick proteins at the feeding site as well as their affinity for their respective host targets. The current review showed that the majority of proteins found in tick saliva or salivary glands thus far characterized will be functional at the tick feeding site. It was also shown how ticks may circumvent the problems presented by an equilibrium system. Even so, inferring functional relevance without estimating concentration or affinity at the feeding site remains a risky endeavor. Future aims in salivary gland biology should focus on quantification of protein concentration secreted during feeding as well as in the actual feeding site. This should provide more accurate estimates of functional relevance.

## Author Contributions

BM conceptualized the study and wrote the manuscript.

## Conflict of Interest Statement

The author declares that the research was conducted in the absence of any commercial or financial relationships that could be construed as a potential conflict of interest.

## References

[B1] Adams T. E. Huntington J. A. (2006). Thrombin–Cofactor interactions. Structural insights into regulatory mechanisms. *Arterioscler. Thromb. Vasc. Biol.* 26 1738–1745. 10.1161/01.atv.0000228844.65168.d1 16728654

[B2] Alenazi Y. Singh K. Davies G. Eaton J. R. O. Elders P. Kawamura A. (2018). Genetically engineered two-warhead evasins provide a method to achieve precision targeting of disease-relevant chemokine subsets. *Sci. Rep.* 8:6333. 10.1038/s41598-018-24568-9 29679010 PMC5910400

[B3] Anastopoulos P. Thurn M. J. Broady K. W. (1991). Anticoagulant in the tick *Ixodes holocyclus*. *Aust. Vet. J.* 68 366–367. 10.1111/j.1751-0813.1991.tb00740.x 1776937

[B4] Andersen J. F. Ribeiro J. M. C. (2017). “Salivary kratagonists: scavengers of host physiological effectors during blood feeding,” in *Arthropod Vector: Controller of Disease Transmission* Vol. 2 eds Wikel S. K. Aksoy S. Dimopoulos G. (Amsterdam: Elsevier) 51–63. 10.1016/b978-0-12-805360-7.00004-6

[B5] Anguita J. Ramamoorthi N. Hovius J. W. Das S. Thomas V. Persinski R. (2002). Salp15, an *Ixodes scapularis* salivary protein, inhibits CD4(+) T cell activation. *Immunity* 16 849–859. 10.1016/s1074-7613(02)00325-4 12121666

[B6] Anisuzzaman M. Hatta T. Miyoshi T. Matsubayashi M. Islam M. K. Alim M. A. (2014). Longistatin in tick saliva blocks advanced glycation end-product receptor activation. *J. Clin. Invest.* 124 4429–4444. 10.1172/jci74917 25401185 PMC4191044

[B7] Anisuzzaman M. Islam K. Alim M. A. Miyoshi T. Hatta T. Yamaji K. (2011). Longistatin, a plasminogen activator, is key to the availability of blood-meals for ixodid ticks. *PLoS Pathog.* 7:e1001312. 10.1371/journal.ppat.1001312 21423674 PMC3053353

[B8] Anisuzzaman M. Islam K. Alim M. A. Miyoshi T. Hatta T. Yamaji K. (2012). Longistatin is an unconventional serine protease and induces protective immunity against tick infestation. *Mol. Biochem. Parasitol.* 182 45–53. 10.1016/j.molbiopara.2011.12.002 22206819

[B9] Anisuzzaman M. Islam K. Miyoshi T. Alim M. A. Hatta T. Yamaji K. (2010). Longistatin, a novel EF-hand protein from the ixodid tick *Haemaphysalis longicornis*, is required for acquisition of host blood-meals. *Int. J. Parasitol.* 40 721–729. 10.1016/j.ijpara.2009.11.004 19968997

[B10] Arcà B. Ribeiro J. M. (2018). Saliva of hematophagous insects: a multifaceted toolkit. *Curr. Opin. Insect Sci.* 29 102–109. 10.1016/j.cois.2018.07.012 30551815

[B11] Arolas J. L. Lorenzo J. Rovira A. Castellà J. Aviles F. X. Sommerhoff C. P. (2005). A carboxypeptidase inhibitor from the tick *Rhipicephalus bursa*: isolation, cDNA cloning, recombinant expression, and characterization. *J. Biol. Chem.* 280 3441–3448. 10.1074/jbc.m411086200 15561703

[B12] Arthur D. R. (1970). Tick feeding and its implications. *Adv. Parasitol.* 8 275–292. 10.1016/s0065-308x(08)60258-4 4327864

[B13] Assumpção T. C. Ma D. Mizurini D. M. Kini R. M. Ribeiro J. M. Kotsyfakis M. (2016). In vitro mode of action and anti-thrombotic activity of boophilin, a multifunctional Kunitz protease inhibitor from the midgut of a tick vector of babesiosis, *Rhipicephalus microplus*. *PLoS Negl. Trop. Dis.* 10:e0004298. 10.1371/journal.pntd.0004298 26745503 PMC4706430

[B14] Assumpção T. C. Mizurini D. M. Ma D. Monteiro R. Q. Ahlstedt S. Reyes M. (2018). Ixonnexin from tick saliva promotes fibrinolysis by interacting with plasminogen and tissue-type plasminogen activator, and prevents arterial thrombosis. *Sci. Rep.* 8:4806. 10.1038/s41598-018-22780-1 29555911 PMC5859130

[B15] Bakkes D. K. De Klerk D. Latif A. A. Mans B. J. (2018). Integrative taxonomy of Afrotropical *Ornithodoros* (*Ornithodoros*) (Acari: Ixodida: Argasidae). *Ticks Tick Borne Dis.* 9 1006–1037. 10.1016/j.ttbdis.2018.03.024 29625921

[B16] Bastiani M. Hillebrand S. Horn F. Kist T. B. Guimarães J. A. Termignoni C. (2002). Cattle tick *Boophilus microplus* salivary gland contains a thiol-activated metalloendopeptidase displaying kininase activity. *Insect Biochem. Mol. Biol.* 32 1439–1446. 10.1016/s0965-1748(02)00064-4 12530211

[B17] Batista I. F. Ramos O. H. Ventura J. S. Junqueira-de-Azevedo I. L. Ho P. L. Chudzinski-Tavassi A. M. (2010). A new factor Xa inhibitor from *Amblyomma cajennense* with a unique domain composition. *Arch. Biochem. Biophys.* 493 151–156. 10.1016/j.abb.2009.10.009 19853573

[B18] Beaufays J. Adam B. Menten-Dedoyart C. Fievez L. Grosjean A. Decrem Y. (2008). Ir-LBP, an *Ixodes ricinus* tick salivary LTB4-binding lipocalin, interferes with host neutrophil function. *PLoS One* 3:e3987. 10.1371/journal.pone.0003987 19096526 PMC2600610

[B19] Binnington K. C. Kemp D. H. (1980). Role of tick salivary glands in feeding and disease transmission. *Adv. Parasitol.* 18 315–339. 10.1016/s0065-308x(08)60403-0 6776790

[B20] Bopp C. Hofer S. Weitz J. Bierhaus A. Nawroth P. P. Martin E. (2008). sRAGE is elevated in septic patients and associated with patients outcome. *J. Surg. Res.* 147 79–83. 10.1016/j.jss.2007.07.014 17981300

[B21] Brahma R. K. Blanchet G. Kaur S. Kini R. M. Doley R. (2017). Expression and characterization of haemathrins, madanin-like thrombin inhibitors, isolated from the salivary gland of tick *Haemaphysalis bispinosa* (Acari: Ixodidae). *Thromb. Res.* 152 20–29. 10.1016/j.thromres.2017.01.012 28213103

[B22] Branco V. G. Iqbal A. Alvarez-Flores M. P. Sciani J. M. de Andrade S. A. Iwai L. K. (2016). Amblyomin-X having a Kunitz-type homologous domain, is a noncompetitive inhibitor of FXa and induces anticoagulation in vitro and in vivo. *Biochim. Biophys. Acta* 1864 1428–1435. 10.1016/j.bbapap.2016.07.011 27479486

[B23] Brown S. J. Knapp F. W. (1980a). *Amblyomma americanum*: sequential histological analysis of larval and nymphal feeding sites on guinea pigs. *Exp. Parasitol.* 49 188–205. 10.1016/0014-4894(80)90116-2 7364007

[B24] Brown S. J. Knapp F. W. (1980b). *Amblyomma americanum*: sequential histological analysis of adult feeding sites on guinea pigs. *Exp. Parasitol.* 49 303–318. 10.1016/0014-4894(80)90067-3 7371734

[B25] Brown S. J. Worms M. J. Askenase P. W. (1983). *Rhipicephalus appendiculatus*: larval feeding sites in guinea pigs actively sensitized and receiving immune serum. *Exp. Parasitol.* 55 111–120. 10.1016/0014-4894(83)90004-8 6822283

[B26] Bullard R. Allen P. Chao C. C. Douglas J. Das P. Morgan S. E. (2016). Structural characterization of tick cement cones collected from in vivo and artificial membrane blood-fed lone star ticks (*Amblyomma americanum*). *Ticks Tick Borne Dis.* 7 880–892. 10.1016/j.ttbdis.2016.04.006 27118479 PMC5460760

[B27] Butenas S. Mann K. G. (2002). Blood coagulation. *Biochemistry* 67 3–12. 11841335 10.1023/a:1013985911759

[B28] Calvo E. Mans B. J. Andersen J. F. Ribeiro J. M. (2006). Function and evolution of a mosquito salivary protein family. *J. Biol. Chem.* 281 1935–1942. 10.1074/jbc.m510359200 16301315

[B29] Cao J. Shi L. Zhou Y. Gao X. Zhang H. Gong H. (2013). Characterization of a new Kunitz-type serine protease inhibitor from the hard tick *Rhipicephalus hemaphysaloides*. *Arch. Insect Biochem. Physiol.* 84 104–113. 10.1002/arch.21118 25708749

[B30] Chalaire K. C. Kim T. K. Garcia-Rodriguez H. Mulenga A. (2011). Amblyomma americanum (L.) (Acari: Ixodidae) tick salivary gland serine protease inhibitor (serpin) 6 is secreted into tick saliva during tick feeding. *J. Exp. Biol.* 214 665–673. 10.1242/jeb.052076 21270316 PMC3027472

[B31] Chand K. K. Lee K. M. Lavidis N. A. Rodriguez-Valle M. Ijaz H. Koehbach J. (2016). Tick holocyclotoxins trigger host paralysis by presynaptic inhibition. *Sci. Rep.* 6:29446. 10.1038/srep29446 27389875 PMC4937380

[B32] Chmelař J. Kotál J. Karim S. Kopacek P. Francischetti I. M. B. Pedra J. H. F. (2016a). Sialomes and mialomes: a systems-biology view of tick tissues and tick-host interactions. *Trends Parasitol.* 32 242–254. 10.1016/j.pt.2015.10.002 26520005 PMC4767689

[B33] Chmelař J. Kotál J. Kopeckı J. Pedra J. H. Kotsyfakis M. (2016b). All for one and one for all on the tick-host battlefield. *Trends Parasitol.* 32 368–377. 10.1016/j.pt.2016.01.004 26830726 PMC4851932

[B34] Chmelař J. Kotál J. Langhansová H. Kotsyfakis M. (2017). Protease inhibitors in tick saliva: the role of serpins and cystatins in tick-host-pathogen interaction. *Front. Cell. Infect. Microbiol.* 7:216. 10.3389/fcimb.2017.00216 28611951 PMC5447049

[B35] Chmelař J. Oliveira C. J. Rezacova P. Francischetti I. M. Kovarova Z. Pejler G. (2011). A tick salivary protein targets cathepsin G and chymase and inhibits host inflammation and platelet aggregation. *Blood* 117 736–744. 10.1182/blood-2010-06-293241 20940421 PMC3031492

[B36] Chou T.-C. Talalay P. (1977). A simple generalized equation for the analysis of multiple inhibition of Michaelis-Mentin kinetics systems. *J. Biol. Chem.* 252 6438–6442. 893418

[B37] Ciprandi A. de Oliveira S. K. Masuda A. Horn F. Termignoni C. (2006). *Boophilus microplus*: its saliva contains microphilin, a small thrombin inhibitor. *Exp. Parasitol.* 114 40–46. 10.1016/j.exppara.2006.02.010 16600217

[B38] Coller B. S. Beer J. H. Scudder L. E. Steinberg M. H. (1989). Collagen-platelet interactions: evidence for a direct interaction of collagen with platelet GPIa/IIa and an indirect interaction with platelet GPIIb/IIIa mediated by adhesive proteins. *Blood* 74 182–192. 2546619

[B39] Copley S. D. (2015). An evolutionary biochemist’s perspective on promiscuity. *Trends Biochem. Sci.* 40 72–78. 10.1016/j.tibs.2014.12.004 25573004 PMC4836852

[B40] Copley S. D. (2017). Shining a light on enzyme promiscuity. *Curr. Opin. Struct. Biol.* 47 167–175. 10.1016/j.sbi.2017.11.001 29169066

[B41] Cornwall J. W. Patton W. S. (1914). Some observations on the salivary secretion of the commoner blood-sucking insects and ticks. *Indian J. Med. Res.* 2 569–593.

[B42] Couvreur B. Beaufays J. Charon C. Lahaye K. Gensale F. Denis V. (2008). Variability and action mechanism of a family of anticomplement proteins in *Ixodes ricinus*. *PLoS One* 3:e1400. 10.1371/journal.pone.0001400 18167559 PMC2151134

[B43] Daix V. Schroeder H. Praet N. Georgin J. P. Chiappino I. Gillet L. (2007). *Ixodes* ticks belonging to the *Ixodes ricinus* complex encode a family of anticomplement proteins. *Insect Mol. Biol.* 16 155–166. 10.1111/j.1365-2583.2006.00710.x 17298559

[B44] Davis T. L. Sharif N. A. (2000). Pharmacological characterization of [3H]-prostaglandin E2 binding to the cloned human EP4 prostanoid receptor. *Br. J. Pharmacol.* 130 1919–1926. 10.1038/sj.bjp.0703525 10952683 PMC1572280

[B45] de Castro M. H. de Klerk D. Pienaar R. Latif A. A. Rees D. J. Mans B. J. (2016). De novo assembly and annotation of the salivary gland transcriptome of *Rhipicephalus appendiculatus* male and female ticks during blood feeding. *Ticks and Tick-borne Dis.* 7 536–548. 10.1016/j.ttbdis.2016.01.014 26830274

[B46] de Castro M. H. de Klerk D. Pienaar R. Rees D. J. G. R. Mans B. J. (2017). Sialotranscriptomics of *Rhipicephalus zambeziensis* reveal intricate expression profiles of secretory proteins and suggest tight temporal transcriptional regulation during blood feeding. *Parasit. Vectors* 10:384. 10.1186/s13071-017-2312-4 PMC5553602 28797301

[B47] de Meillon B. (1942). A toxin from the eggs of South African ticks. *S. Afr. J. Med. Sci.* 7 226–235.

[B48] Decrem Y. Rath G. Blasioli V. Cauchie P. Robert S. Beaufays J. (2009). Ir-CPI, a coagulation contact phase inhibitor from the tick *Ixodes ricinus*, inhibits thrombus formation without impairing hemostasis. *J. Exp. Med.* 206 2381–2395. 10.1084/jem.20091007 19808248 PMC2768864

[B49] Delvaeye M. Conway E. M. (2009). Coagulation and innate immune responses: can we view them separately? *Blood* 114 2367–2374. 10.1182/blood-2009-05-199208 19584396

[B50] Deppermann C. Kubes P. (2016). Platelets and infection. *Semin. Immunol.* 28 536–545. 10.1016/j.smim.2016.10.005 27769639

[B51] Déruaz M. Bonvin P. Severin I. C. Johnson Z. Krohn S. Power C. A. (2013). Evasin-4, a tick-derived chemokine-binding protein with broad selectivity can be modified for use in preclinical disease models. *FEBS J.* 280 4876–4887. 10.1111/febs.12463 23910450 PMC4240464

[B52] Déruaz M. Frauenschuh A. Alessandri A. L. Dias J. M. Coelho F. M. Russo R. C. (2008). Ticks produce highly selective chemokine binding proteins with antiinflammatory activity. *J. Exp. Med.* 205 2019–2031. 10.1084/jem.20072689 18678732 PMC2526197

[B53] Dharampaul S. Kaufman W. R. Belosevic M. (1993). Differential recognition of saliva antigens from the ixodid tick *Amblyomma hebraeum* (Acari: Ixodidae) by sera from infested and immunized rabbits. *J. Med. Entomol.* 30 262–266. 10.1093/jmedent/30.1.262 8433335

[B54] Díaz-Martín V. Manzano-Román R. Oleaga A. Encinas-Grandes A. Pérez-Sánchez R. (2013a). Cloning and characterization of a plasminogen-binding enolase from the saliva of the argasid tick *Ornithodoros moubata*. *Vet. Parasitol.* 191 301–314. 10.1016/j.vetpar.2012.09.019 23089148

[B55] Díaz-Martín V. Manzano-Román R. Valero L. Oleaga A. Encinas-Grandes A. Pérez-Sánchez R. (2013b). An insight into the proteome of the saliva of the argasid tick *Ornithodoros moubata* reveals important differences in saliva protein composition between the sexes. *J. Proteomics* 80 216–235. 10.1016/j.jprot.2013.01.015 23416086

[B56] Dickinson R. G. O’Hagan J. E. Schotz M. Binnington K. C. Hegarty M. P. (1976). Prostaglandin in the saliva of the cattle tick *Boophilus microplus*. *Aust. J. Exp. Biol. Med. Sci.* 54 475–486. 1021092 10.1038/icb.1976.48

[B57] Dorn G. W. II DeJesus A. (1991). Human platelet aggregation and shape change are coupled to separate thromboxane A2-prostaglandin H2 receptors. *Am. J. Physiol.* 260 H327–H334. 1825455 10.1152/ajpheart.1991.260.2.H327

[B58] Drazen J. M. Austen K. F. Lewis R. A. Clark D. A. Goto G. Marfat A. (1980). Comparative airway and vascular activities of leukotrienes C-1 and D *in vivo* and *in vitro*. *Proc. Natl. Acad. Sci. U.S.A.* 77 4354–4358. 6933488 10.1073/pnas.77.7.4354 PMC349833

[B59] Eaton J. R. O. Alenazi Y. Singh K. Davies G. Geis-Asteggiante L. Kessler B. (2018). The N-terminal domain of a tick evasin is critical for chemokine binding and neutralization and confers specific binding activity to other evasins. *J. Biol. Chem.* 293 6134–6146. 10.1074/jbc.RA117.000487 29487134 PMC5912465

[B60] Ehebauer M. T. Mans B. J. Gaspar A. R. Neitz A. W. (2002). Identification of extrinsic blood coagulation pathway inhibitors from the tick *Ornithodoros savignyi* (Acari: Argasidae). *Exp. Parasitol.* 101 138–148. 10.1016/s0014-4894(02)00102-9 12427468

[B61] Elgin M. (2010). “Reductionism in biology: an example of biochemistry,” in *The Present Situation in the Philosophy of Science. The Philosophy of Science in a European Perspective* Vol. 1 ed. Stadler F. (Dortrecht: Spinger).

[B62] Faas M. M. Sáez T. de Vos P. (2017). Extracellular ATP and adenosine: the Yin and Yang in immune responses? *Mol. Aspects. Med.* 55 9–19. 10.1016/j.mam.2017.01.002 28093236

[B63] Ferrer-Lopez P. Renesto P. Schattner M. Bassot S. Laurent P. Chignard M. (1990). Activation of human platelets by C5a-stimulated neutrophils: a role for cathepsin G. *Am. J. Physiol.* 258 C1100–C1107. 2360620 10.1152/ajpcell.1990.258.6.C1100

[B64] Feyerabend P. (1975). *Against Method: Outline of an Anarchistic Theory of Knowledge.* Brooklyn, NY: Verso Books, 339.

[B65] Figueiredo A. C. de Sanctis D. Pereira P. J. (2013). The tick-derived anticoagulant madanin is processed by thrombin and factor Xa. *PLoS One* 8:e71866. 10.1371/journal.pone.0071866 23951260 PMC3741208

[B66] Flower D. R. (1996). The lipocalin protein family: structure and function. *Biochem. J.* 318 1–14. 10.1042/bj3180001 8761444 PMC1217580

[B67] Francischetti I. M. Mans B. J. Meng Z. Gudderra N. Veenstra T. D. Pham V. M. (2008). An insight into the sialome of the soft tick, *Ornithodorus parkeri*. *Insect Biochem. Mol. Biol.* 38 1–21. 10.1016/j.ibmb.2007.09.009 18070662 PMC2233652

[B68] Francischetti I. M. Mather T. N. Ribeiro J. M. (2003). Cloning of a salivary gland metalloprotease and characterization of gelatinase and fibrin(ogen)lytic activities in the saliva of the Lyme disease tick vector *Ixodes scapularis*. *Biochem. Biophys. Res. Commun.* 305 869–875. 10.1016/s0006-291x(03)00857-x 12767911 PMC2903890

[B69] Francischetti I. M. Mather T. N. Ribeiro J. M. (2004). Penthalaris, a novel recombinant five-Kunitz tissue factor pathway inhibitor (TFPI) from the salivary gland of the tick vector of Lyme disease, *Ixodes scapularis*. *Thromb. Haemost.* 91 886–898. 10.1160/th03-11-0715 15116248

[B70] Francischetti I. M. Mather T. N. Ribeiro J. M. (2005). Tick saliva is a potent inhibitor of endothelial cell proliferation and angiogenesis. *Thromb. Haemost.* 94 167–174. 10.1160/th04-09-0566 16113800 PMC2893037

[B71] Francischetti I. M. Sa-Nunes A. Mans B. J. Santos I. M. Ribeiro J. M. (2009). The role of saliva in tick feeding. *Front. Biosci.* 14:2051–2088. 10.2741/3363 PMC2785505 19273185

[B72] Francischetti I. M. Valenzuela J. G. Andersen J. F. Mather T. N. Ribeiro J. M. (2002). Ixolaris, a novel recombinant tissue factor pathway inhibitor (TFPI) from the salivary gland of the tick, *Ixodes scapularis*: identification of factor X and factor Xa as scaffolds for the inhibition of factor VIIa/tissue factor complex. *Blood* 99 3602–3612. 10.1182/blood-2001-12-0237 11986214

[B73] Frauenschuh A. Power C. A. Déruaz M. Ferreira B. R. Silva J. S. Teixeira M. M. (2007). Molecular cloning and characterization of a highly selective chemokine-binding protein from the tick *Rhipicephalus sanguineus*. *J. Biol. Chem.* 282 27250–27258. 10.1074/jbc.m704706200 17640866

[B74] Frojmovic M. M. Mooney R. F. Wong T. (1994). Dynamics of platelet glycoprotein IIb-IIIa receptor expression and fibrinogen binding. I. Quantal activation of platelet subpopulations varies with adenosine diphosphate concentration. *Biophys. J.* 67 2060–2068. 10.1016/s0006-3495(94)80689-7 7858143 PMC1225581

[B75] Garg R. Juncadella I. J. Ramamoorthi N. Ashish Ananthanarayanan S. K. Thomas V. Rincón M. (2006). Cutting edge: CD4 is the receptor for the tick saliva immunosuppressor, Salp15. *J. Immunol.* 177 6579–6583. 10.4049/jimmunol.177.10.6579 17082567 PMC4302324

[B76] Gaspar A. R. Crause J. C. Neitz A. W. (1995). Identification of anticoagulant activities in the salivary glands of the soft tick, *Ornithodoros savignyi*. *Exp. Appl. Acarol.* 19 117–127. 10.1007/bf00052551 7656730

[B77] Gaspar A. R. Joubert A. M. Crause J. C. Neitz A. W. (1996). Isolation and characterization of an anticoagulant from the salivary glands of the tick, *Ornithodoros savignyi* (Acari: Argasidae). *Exp. Appl. Acarol.* 20 583–598. 10.1007/bf00052809 8952072

[B78] Ghiglieri-Bertez C. Cristol J. P. Bonne C. (1986). High-affinity binding site for leukotriene C4 in human erythrocytes. *Biochim. Biophys. Acta* 879 97–102. 10.1016/0005-2760(86)90271-7 3021227

[B79] Giles T. C. Emes R. D. (2017). Inferring function from homology. *Meth. Mol. Biol.* 1526 23–40. 10.1007/978-1-4939-6613-4_2 27896734

[B80] Gong H. Zhou J. Liao M. Hatta T. Harnnoi T. Umemiya R. (2007). Characterization of a carboxypeptidase inhibitor from the tick *Haemaphysalis longicornis*. *J. Insect Physiol.* 53 1079–1087. 10.1016/j.jinsphys.2007.06.008 17651749

[B81] Gregson J. D. (1973). *Tick Paralysis: An Appraisal of Natural and Experimental Data.* Ottawa, ON: Information Division, Canada Department of Agriculture. 10.1016/j.jinsphys.2007.06.008

[B82] Grunclová L. Horn M. Vancová M. Sojka D. Franta Z. Mares M. (2006). Two secreted cystatins of the soft tick *Ornithodoros moubata*: differential expression pattern and inhibitory specificity. *Biol Chem.* 387 1635–1644. 17132111 10.1515/BC.2006.204

[B83] Hackenberg M. Kotsyfakis M. (2018). Exosome-mediated pathogen transmission by arthropod vectors. *Trends Parasitol.* 34 549–552. 10.1016/j.pt.2018.04.001 29703586

[B84] Hackenberg M. Langenberger D. Schwarz A. Erhart J. Kotsyfakis M. (2017). In silico target network analysis of *de novo*-discovered, tick saliva-specific microRNAs reveals important combinatorial effects in their interference with vertebrate host physiology. *RNA* 23 1259–1269. 10.1261/rna.061168.117 28473453 PMC5513070

[B85] Hamberg M. Svensson J. Samuelsson B. (1975). Thromboxanes: a new group of biologically active compounds derived from prostaglandin endoperoxides. *Proc. Natl. Acad. Sci. U.S.A.* 72 2994–2998. 10.1073/pnas.72.8.2994 1059088 PMC432905

[B86] Haycraft J. B. (1884). Ueber die Einwirkung eines Sekretes des officinellen Blutegels auf die Gerinnbarkeit des Blutes. *Arch. für exp. Pathol. u. Pharmakol.* 18:209 10.1007/bf01833843

[B87] Hayward J. Sanchez J. Perry A. Huang C. Rodriguez Valle M. Canals M. (2018). Ticks from diverse genera encode chemokine-inhibitory evasin proteins. *J. Biol. Chem.* 292 15670–15680. 10.1074/jbc.m117.807255 28778927 PMC5612101

[B88] Heller C. (1858). Zur anatomie von *Argas persicus*. *Sitzungberichte d. Kaiserl. Akad. Wien* 30 297–326.

[B89] Hepburn N. J. Williams A. S. Nunn M. A. Chamberlain-Banoub J. C. Hamer J. Morgan B. P. (2007). In vivo characterization and therapeutic efficacy of a C5-specific inhibitor from the soft tick *Ornithodoros moubata*. *J. Biol. Chem.* 282 8292–8299. 10.1074/jbc.m609858200 17215252

[B90] Hoeppli R. Feng L. C. (1933). Experimental studies on ticks. *Chin. Med. J.* 47 29–43.

[B91] Hoffmann A. Walsmann P. Riesener G. Paintz M. Markwardt F. (1991). Isolation and characterization of a thrombin inhibitor from the tick *Ixodes ricinus*. *Pharmazie* 46 209–212. 1881945

[B92] Horn F. dos Santos P. C. Termignoni C. (2000). *Boophilus microplus* anticoagulant protein: an antithrombin inhibitor isolated from the cattle tick saliva. *Arch. Biochem. Biophys.* 384 68–73. 10.1006/abbi.2000.2076 11147837

[B93] Hovius J. W. de Jong M. A. den Dunnen J. Litjens M. Fikrig E. van der Poll T. (2008). Salp15 binding to DC-SIGN inhibits cytokine expression by impairing both nucleosome remodeling and mRNA stabilization. *PLoS Pathog.* 4:e31. 10.1371/journal.ppat.0040031 18282094 PMC2242833

[B94] Howell C. J. (1966). Collection of salivary gland secretion from the argasid *Ornithodoros savignyi* (Audouin) (1827) by the use of a pharmacological stimulant. *J. S. Afr. Vet. Med. Assoc.* 37 236–239.

[B95] Howell C. J. Neitz A. W. H. Potgieter D. J. J. (1975). Some toxic and chemical properties of the oral secretion of the sand tampan, *Ornithodoros savignyi* (Audouin) (1827). *Onderstepoort J. Vet. Res.* 43 99–102. 1196584

[B96] Hu G. Drescher K. M. Chen X. M. (2012). Exosomal miRNAs: biological properties and therapeutic potential. *Front. Genet.* 3:56 10.3389/fgene.2012.00056 PMC3330238 22529849

[B97] Ibelli A. M. Kim T. K. Hill C. C. Lewis L. A. Bakshi M. Miller S. (2014). A blood meal-induced *Ixodes scapularis* tick saliva serpin inhibits trypsin and thrombin, and interferes with platelet aggregation and blood clotting. *Int. J. Parasitol.* 44 369–379. 10.1016/j.ijpara.2014.01.010 24583183 PMC4089096

[B98] Ibrahim M. A. Ghazy A. H. Maharem T. M. Khalil M. I. (2001a). Factor Xa (FXa) inhibitor from the nymphs of the camel tick *Hyalomma dromedarii*. *Comp. Biochem. Physiol. B Biochem. Mol. Biol.* 130 501–512. 10.1016/s1096-4959(01)00459-6 11691627

[B99] Ibrahim M. A. Ghazy A. H. Maharem T. Khalil M. (2001b). Isolation and properties of two forms of thrombin inhibitor from the nymphs of the camel tick *Hyalomma dromedarii* (Acari: Ixodidae). *Exp. Appl. Acarol.* 25 675–698. 12171275 10.1023/a:1016136207308

[B100] Ibrahim M. A. Masoud H. M. M. (2018). Thrombin inhibitor from the salivary gland of the camel tick *Hyalomma dromedarii*. *Exp. Appl. Acarol.* 74 85–97. 10.1007/s10493-017-0196-9 29255966

[B101] Imamura S. da Silva Vaz Junior I. Sugino M. Ohashi K. Onuma M. (2005). A serine protease inhibitor (serpin) from *Haemaphysalis longicornis* as an anti-tick vaccine. *Vaccine* 23 1301–1311. 10.1016/j.vaccine.2004.08.041 15652673

[B102] Inokuma H. Kemp D. H. Willadsen P. (1994). Comparison of prostaglandin E2 (PGE2) in salivary gland of *Boophilus microplus*, *Haemaphysalis longicornis* and *Ixodes holocyclus*, and quantification of PGE2 in saliva, hemolymph, ovary and gut of *B. microplus*. *J. Vet. Med. Sci.* 56 1217–1218. 10.1292/jvms.56.1217 7696426

[B103] Iqbal A. Goldfeder M. B. Marques-Porto R. Asif H. Souza J. G. Faria F. (2017). Revisiting antithrombotic therapeutics; sculptin, a novel specific, competitive, reversible, scissile and tight binding inhibitor of thrombin. *Sci. Rep.* 7:1431. 10.1038/s41598-017-01486-w 28469161 PMC5431157

[B104] Iwanaga S. Isawa H. Yuda M. (2014). Horizontal gene transfer of a vertebrate vasodilatory hormone into ticks. *Nature Comm.* 5:3373. 10.1038/ncomms4373 24556716

[B105] Iwanaga S. Okada M. Isawa H. Morita A. Yuda M. Chinzei Y. (2003). Identification and characterization of novel salivary thrombin inhibitors from the ixodidae tick, *Haemaphysalis longicornis*. *Eur. J. Biochem.* 270 1926–1934. 10.1046/j.1432-1033.2003.03560.x 12709051

[B106] Iyer J. K. Koh C. Y. Kazimirova M. Roller L. Jobichen C. Swaminathan K. (2017). Avathrin: a novel thrombin inhibitor derived from a multicopy precursor in the salivary glands of the ixodid tick, *Amblyomma variegatum*. *FASEB J.* 31 2981–2995. 10.1096/fj.201601216R 28363953

[B107] Jablonka W. Kotsyfakis M. Mizurini D. M. Monteiro R. Q. Lukszo J. Drake S. K. (2015). Identification and mechanistic analysis of a novel tick-derived inhibitor of Thrombin. *PLoS One* 10:e0133991. 10.1371/journal.pone.0133991 26244557 PMC4526366

[B108] Jore M. M. Johnson S. Sheppard D. Barber N. M. Li Y. I. Nunn M. A. (2016). Structural basis for therapeutic inhibition of complement C5. *Nat. Struct. Mol. Biol.* 23 378–386. 10.1038/nsmb.3196 27018802 PMC5771465

[B109] Jung S. M. Moroi M. (1998). Platelets interact with soluble and insoluble collagens through characteristically different reactions. *J. Biol. Chem.* 273 14827–14837. 10.1074/jbc.273.24.14827 9614084

[B110] Karczewski J. Endris R. Connolly T. M. (1994). Disagregin is a fibrinogen receptor antagonist lacking the Arg-Gly-Asp sequence from the tick, *Ornithodoros moubata*. *J. Biol. Chem.* 269 6702–6708. 8120028

[B111] Karczewski J. Waxman L. Endris R. G. Connolly T. M. (1995). An inhibitor from the argasid tick *Ornithodoros moubata* of cell adhesion to collagen. *Biochem. Biophys. Res. Commun.* 208 532–541. 10.1006/bbrc.1995.1371 7695604

[B112] Kato N. Iwanaga S. Okayama T. Isawa H. Yuda M. Chinzei Y. (2005). Identification and characterization of the plasma kallikrein-kinin system inhibitor, haemaphysalin, from hard tick, *Haemaphysalis longicornis*. *Thromb. Haemost.* 93 359–367. 10.1160/th04-05-0319 15711755

[B113] Kazimírová M. Jancinová V. Petríková M. Takác P. Labuda M. Nosál R. (2002). An inhibitor of thrombin-stimulated blood platelet aggregation from the salivary glands of the hard tick *Amblyomma variegatum* (Acari: Ixodidae). *Exp. Appl. Acarol.* 28 97–105. 10.1007/978-94-017-3526-1_7 14570120

[B114] Keller P. M. Waxman L. Arnold B. A. Schultz L. D. Condra C. Connolly T. M. (1993). Cloning of the cDNA and expression of moubatin, an inhibitor of platelet aggregation. *J. Biol. Chem.* 268 5450–5456. 8449907

[B115] Kemp D. H. Stone B. F. Binnington K. C. (1982). “Tick attachment and feeding: role of the mouthparts, feeding apparatus, salivary gland secretions and the host response,” in *Physiology of Ticks*, eds Obenchain F. D. Galun R. (Oxford: Pergamon Press).

[B116] Kim T. K. Tirloni L. Pinto A. F. Moresco J. Yates J. R. III da Silva Vaz I. Jr. (2017). *Ixodes scapularis* tick saliva proteins sequentially secreted every 24 h during blood feeding. *PLoS Negl. Trop. Dis.* 10:e0004323. 10.1371/journal.pntd.0004323 26751078 PMC4709002

[B117] Kim T. K. Tirloni L. Radulovic Z. Lewis L. Bakshi M. Hill C. (2015). Conserved *Amblyomma americanum* tick serpin19, an inhibitor of blood clotting factors Xa and XIa, trypsin and plasmin, has anti-haemostatic functions. *Int. J. Parasitol.* 45 613–627. 10.1016/j.ijpara.2015.03.009 25957161 PMC4490099

[B118] Kiszewski A. E. Matuschka F. R. Spielman A. (2001). Mating strategies and spermiogenesis in ixodid ticks. *Annu. Rev. Entomol.* 46 167–182. 11112167 10.1146/annurev.ento.46.1.167

[B119] Koh C. Y. Kazimirova M. Trimnell A. Takac P. Labuda M. Nuttall P. A. (2007). Variegin, a novel fast and tight binding thrombin inhibitor from the tropical bont tick. *J. Biol. Chem.* 282 29101–29113. 10.1074/jbc.m705600200 17684009

[B120] Kotál J. Stergiou N. Buša M. Chlastáková A. Beránková Z. Řezáčová P. (2019). The structure and function of Iristatin, a novel immunosuppressive tick salivary cystatin. *Cell Mol. Life Sci.* 30747251 10.1007/s00018-019-03034-3 PMC11105445

[B121] Kotsyfakis M. Karim S. Andersen J. F. Mather T. N. Ribeiro J. M. (2007). Selective cysteine protease inhibition contributes to blood-feeding success of the tick *Ixodes scapularis*. *J. Biol. Chem.* 282 29256–29263. 10.1074/jbc.m703143200 17698852

[B122] Kotsyfakis M. Sá-Nunes A. Francischetti I. M. Mather T. N. Andersen J. F. Ribeiro J. M. (2006). Antiinflammatory and immunosuppressive activity of sialostatin L, a salivary cystatin from the tick *Ixodes scapularis*. *J. Biol. Chem.* 281 26298–26307. 10.1074/jbc.m513010200 16772304

[B123] Krilis S. Lewis R. A. Corey E. J. Austen K. F. (1983). Specific receptors for leukotriene C4 on a smooth muscle cell line. *J. Clin. Invest.* 72 1516–1519. 10.1172/jci111109 6313763 PMC370437

[B124] Kröber T. Geurin P. M. (2007). In vitro feeding assays for hard ticks. *Trends Parasitol.* 23 445–449. 10.1016/j.pt.2007.07.010 17681859

[B125] Kuhn N. Schmidt C. Q. Schlapschy M. Skerra A. (2016). PASylated Coversin, a C5-specific complement inhibitor with extended pharmacokinetics, shows enhanced anti-hemolytic activity *in vitro*. *Bioconjug. Chem.* 27 2359–2371. 10.1021/acs.bioconjchem.6b00369 27598771

[B126] Kuriyan J. Konforti B. Wemmer D. (2013). *The Molecules of Life: Physical and Chemical Principles.* Routledge: Garland Science, Taylor & Francis Group, 1008.

[B127] Law J. H. Ribeiro J. M. C. Wells M. A. (1992). Biochemical insights derived from insect diversity. *Ann. Rev. Biochem.* 64 87–111. 10.1146/annurev.biochem.61.1.87 1497325

[B128] Leboulle G. Crippa M. Decrem Y. Mejri N. Brossard M. Bollen A. (2002). Characterization of a novel salivary immunosuppressive protein from *Ixodes ricinus* ticks. *J. Biol. Chem.* 277 10083–10089. 11792703 10.1074/jbc.M111391200

[B129] Lewis R. A. Mencia-Huerta J. M. Soberman R. J. Hoover D. Marfat A. Corey E. J. (1982). Radioimmunoassay for leukotriene B4. *Proc. Natl. Acad. Sci. U.S.A.* 79 7904–7908. 6296855 10.1073/pnas.79.24.7904 PMC347458

[B130] Leydig F. (1855). Zum feineren Bau der Arthropoden. *Müller’s Archiv. f. Anat. Physiol. u. wiss. Med.* 1855 376–480.

[B131] Limo M. K. Voigt W. P. Tumbo-Oeri A. G. Njogu R. M. ole-MoiYoi O. K. (1991). Purification and characterization of an anticoagulant from the salivary glands of the ixodid tick *Rhipicephalus appendiculatus*. *Exp. Parasitol.* 72 418–429. 10.1016/0014-4894(91)90088-e 2026216

[B132] Liu Y. H. Xu C. H. Liu Z. G. Liang J. G. Lai R. (2005). Isolation and purification of an inhibitor on platelet aggregation from *Ixodes sinensis*. *Zhongguo Ji Sheng Chong Xue Yu Ji Sheng Chong Bing Za Zhi* 23 424–427. 16566211

[B133] Macedo-Ribeiro S. Almeida C. Calisto B. M. Friedrich T. Mentele R. Stürzebecher J. (2008). Isolation, cloning and structural characterisation of boophilin, a multifunctional Kunitz-type proteinase inhibitor from the cattle tick. *PLoS One* 3:e1624. 10.1371/journal.pone.0001624 18286181 PMC2230226

[B134] MacGlashan D. Jr. (2003). Histamine: a mediator of inflammation. *J. Allergy Clin. Immunol.* 112 S53–S59. 14530789 10.1016/s0091-6749(03)01877-3

[B135] MacGregor I. R. Prowse C. V. (1983). Tissue plasminogen activator in human plasma measured by radioimmunoassay. *Thromb. Res.* 31 461–474. 10.1016/0049-3848(83)90410-3 20218002

[B136] Macpherson A. Liu X. Dedi N. Kennedy J. Carrington B. Durrant O. (2018). The rational design of affinity-attenuated OmCI for the purification of complement C5. *J. Biol. Chem.* 293 14112–14121. 10.1074/jbc.RA118.004043 30030376 PMC6130949

[B137] Mans B. J. (1997). *Biochemical Properties of a Platelet Aggregation Inhibitor of the tick, Ornithodoros savignyi.* . 10.1074/jbc.ra118.004043 30030376 PMC6130949

[B138] Mans B. J. (2005). Tick histamine-binding proteins and related lipocalins: potential as therapeutic agents. *Curr. Opin. Investig. Drugs* 6 1131–1135. 16312134

[B139] Mans B. J. (2011). Evolution of vertebrate hemostatic and inflammatory control mechanisms in blood-feeding arthropods. *J. Innate Immun.* 3 41–51. 10.1159/000321599 20980728

[B140] Mans B. J. (2016). “Glandular matrices and secretions: blood- feeding arthropods,” in *Extracellular Composite Matrices in Arthropods*, eds Cohen E. Moussian B. (Switzerland: Springer), 625–688. 10.1007/978-3-319-40740-1_17

[B141] Mans B. J. Andersen J. F. Schwan T. G. Ribeiro J. M. (2008a). Characterization of anti-hemostatic factors in the argasid, *Argas monolakensis*: implications for the evolution of blood-feeding in the soft tick family. *Insect Biochem. Mol. Biol.* 38 22–41. 10.1016/j.ibmb.2007.09.002 18070663 PMC4274796

[B142] Mans B. J. Ribeiro J. M. Andersen J. F. (2008b). Structure, function, and evolution of biogenic amine-binding proteins in soft ticks. *J. Biol. Chem.* 283 18721–18733. 10.1074/jbc.M800188200 18445596 PMC2441560

[B143] Mans B. J. Andersen J. F. Francischetti I. M. Valenzuela J. G. Schwan T. G. Pham V. M. (2008c). Comparative sialomics between hard and soft ticks: implications for the evolution of blood-feeding behavior. *Insect Biochem. Mol. Biol.* 38 42–58. 10.1016/j.ibmb.2007.09.003 18070664 PMC2211429

[B144] Mans B. J. Coetzee J. Louw A. I. Gaspar A. R. Neitz A. W. (2000). Disaggregation of aggregated platelets by apyrase from the tick, *Ornithodoros savignyi* (Acari: Argasidae). *Exp. Appl. Acarol.* 24 271–282. 11110238 10.1023/a:1006440714276

[B145] Mans B. J. de Castro M. H. Pienaar R. de Klerk D. Gaven P. Genu S. (2016). Ancestral reconstruction of tick lineages. *Ticks Tick Borne Dis.* 7 509–535. 10.1016/j.ttbdis.2016.02.002 26868413

[B146] Mans B. J. Featherston J. de Castro M. H. Pienaar R. (2017). Gene duplication and protein evolution in tick-host interactions. *Front. Cell Infect. Microbiol.* 7:413 10.3389/fcimb.2017.00413 PMC5622192 28993800

[B147] Mans B. J. Gaspar A. R. Louw A. I. Neitz A. W. (1998a). Apyrase activity and platelet aggregation inhibitors in the tick *Ornithodoros savignyi* (Acari: Argasidae). *Exp. Appl. Acarol.* 22 353–366. 9652096 10.1023/a:1024517209621

[B148] Mans B. J. Gasper A. R. Louw A. I. Neitz A. W. (1998b). Purification and characterization of apyrase from the tick, *Ornithodoros savignyi*. *Comp. Biochem. Physiol. B Biochem. Mol. Biol.* 120 617–624. 10.1016/s0305-0491(98)10061-5 14598857

[B149] Mans B. J. Venter J. D. Vrey P. J. Louw A. I. Neitz A. W. (2001). Identification of putative proteins involved in granule biogenesis of tick salivary glands. *Electrophoresis* 22 1739–1746. 10.1002/1522-2683(200105)22:9<1739::aid-elps1739>3.0.co;2-7 11425229

[B150] Mans B. J. Louw A. I. Neitz A. W. (2002a). Disaggregation of aggregated platelets by savignygrin, a αIIbβ3 antagonist from *Ornithodoros savignyi*. *Exp. Appl. Acarol.* 27 231–239. 12593588 10.1023/a:1021613001297

[B151] Mans B. J. Louw A. I. Neitz A. W. (2002b). Savignygrin, a platelet aggregation inhibitor from the soft tick *Ornithodoros savignyi*, presents the RGD integrin recognition motif on the Kunitz-BPTI fold. *J. Biol. Chem.* 277 21371–22378. 11932256 10.1074/jbc.M112060200

[B152] Mans B. J. Louw A. I. Neitz A. W. (2003a). The influence of tick behavior, biotope and host specificity on concerted evolution of the platelet aggregation inhibitor savignygrin, from the soft tick *Ornithodoros savignyi*. *Insect Biochem. Mol. Biol.* 33 623–629. 10.1016/s0965-1748(03)00047-x 12770580

[B153] Mans B. J. Louw A. I. Neitz A. W. (2003b). The major tick salivary gland proteins and toxins from the soft tick, *Ornithodoros savignyi*, are part of the tick Lipocalin family: implications for the origins of tick toxicoses. *Mol. Biol. Evol.* 20 1158–1167. 10.1093/molbev/msg126 12777525

[B154] Mans B. J. Neitz A. W. (2004a). Adaptation of ticks to a blood-feeding environment: evolution from a functional perspective. *Insect Biochem. Mol. Biol.* 34 1–17. 10.1016/j.ibmb.2003.09.002 14723893

[B155] Mans B. J. Neitz A. W. (2004b). Molecular crowding as a mechanism for tick secretory granule biogenesis. *Insect Biochem. Mol. Biol.* 34 1187–1193. 10.1016/j.ibmb.2004.07.007 15522614

[B156] Mans B. J. Neitz A. W. (2004c). The mechanism of αIIbβ3 antagonism by savignygrin and its implications for the evolution of anti-hemostatic strategies in soft ticks. *Insect Biochem. Mol. Biol.* 34 573–584. 10.1016/s0965-1748(04)00038-4 15147758

[B157] Mans B. J. Gothe R. Neitz A. W. (2004a). Biochemical perspectives on paralysis and other forms of toxicoses caused by ticks. *Parasitology* 129 S95–S111. 15938507 10.1017/s0031182003004670

[B158] Mans B. J. Venter J. D. Coons L. B. Louw A. I. Neitz A. W. H. (2004b). A reassessment of argasid tick salivary gland ultrastructure from an immuno-cytochemical perspective. *Exp. Appl. Acarol.* 33 119–129. 10.1023/b 15285144

[B159] Mans B. J. Ribeiro J. M. (2008a). A novel clade of cysteinyl leukotriene scavengers in soft ticks. *Insect Biochem. Mol. Biol.* 38 862–870. 10.1016/j.ibmb.2008.06.002 18675910 PMC2583325

[B160] Mans B. J. Ribeiro J. M. (2008b). Function, mechanism and evolution of the moubatin-clade of soft tick lipocalins. *Insect Biochem. Mol. Biol.* 38 841–852. 10.1016/j.ibmb.2008.06.007 18694828 PMC2613973

[B161] McSwain J. L. Essenberg R. C. Sauer J. R. (1982). Protein changes in the salivary glands of the female lone star tick, *Amblyomma americanum*, during feeding. *J. Parasitol.* 68 100–106. 7077436

[B162] Monteiro R. Q. Rezaie A. R. Ribeiro J. M. Francischetti I. M. (2005). Ixolaris: a factor Xa heparin-binding exosite inhibitor. *Biochem. J.* 387 871–877. 10.1042/bj20041738 15617517 PMC1135020

[B163] Moore J. E. Jr. Brook B. S. Nibbs R. J. B. (2018). Chemokine transport dynamics and emerging recognition of their role in immune function. *Curr. Opin. Biomed. Eng.* 5 90–95. 10.1016/j.cobme.2018.03.001 30320240 PMC6176735

[B164] Motoyashiki T. Tu A. T. Azimov D. A. Ibragim K. (2003). Isolation of anticoagulant from the venom of tick, *Boophilus calcaratus*, from Uzbekistan. *Thromb. Res.* 110 235–241. 10.1016/s0049-3848(03)00409-2 14512088

[B165] Mulenga A. Kim T. Ibelli A. M. (2013). *Amblyomma americanum* tick saliva serine protease inhibitor 6 is a cross-class inhibitor of serine proteases and papain-like cysteine proteases that delays plasma clotting and inhibits platelet aggregation. *Insect Mol. Biol.* 22 306–319. 10.1111/imb.12024 23521000 PMC4058330

[B166] Mulenga A. Macaluso K. R. Simser J. A. Azad A. F. (2003). The American dog tick, *Dermacentor variabilis*, encodes a functional histamine release factor homolog. *Insect Biochem. Mol. Biol.* 33 911–919. 10.1016/s0965-1748(03)00097-3 12915182

[B167] Nakajima C. Imamura S. Konnai S. Yamada S. Nishikado H. Ohashi K. (2006). A novel gene encoding a thrombin inhibitory protein in a cDNA library from *Haemaphysalis longicornis* salivary gland. *J. Vet. Med Sci.* 68 447–452. 10.1292/jvms.68.447 16757887

[B168] Narasimhan S. Koski R. A. Beaulieu B. Anderson J. F. Ramamoorthi N. Kantor F. (2002). A novel family of anticoagulants from the saliva of *Ixodes scapularis*. *Insect Mol. Biol.* 11 641–650. 10.1046/j.1365-2583.2002.00375.x 12421422

[B169] Neelakanta G. Sultana H. Sonenshine D. E. Andersen J. F. (2018). Identification and characterization of a histamine-binding lipocalin-like molecule from the relapsing fever tick *Ornithodoros turicata*. *Insect Mol. Biol.* 27 177–187. 10.1111/imb.12362 29164729

[B170] Neitz A. W. Howell C. J. Potgieter D. J. Bezuidenhout J. D. (1978). Proteins and free amino acids in the salivary secretion and haemolymph of the tick *Amblyomma hebraeum*. *Onderstepoort J. Vet. Res.* 45 235–240. 754123

[B171] Neitz A. W. Prozesky L. Bezuidenhout J. D. Putterill J. F. Potgieter D. J. (1981). An investigation into the toxic principle in eggs of the tick *Amblyomma hebraeum*. *Onderstepoort J. Vet. Res.* 48 109–117. 7312303

[B172] Neitz A. W. H. Howell C. J. Potgieter D. J. J. (1969). Purification of the toxic component in the oral secretion of the sand tampan *Ornithodoros savignyi* (Audouin) (1827). *J. S. Afr. Chem. Ind.* 22 142–149.

[B173] Nielsen H. (2017). Predicting secretory proteins with SignalP. *Methods Mol. Biol.* 1611 59–73. 10.1007/978-1-4939-7015-5_6 28451972

[B174] Nienaber J. Gaspar A. R. Neitz A. W. (1999). Savignin, a potent thrombin inhibitor isolated from the salivary glands of the tick *Ornithodoros savignyi* (Acari: Argasidae). *Exp. Parasitol.* 93 82–91. 10.1006/expr.1999.4448 10502470

[B175] Nunn M. A. Sharma A. Paesen G. C. Adamson S. Lissina O. Willis A. C. (2005). Complement inhibitor of C5 activation from the soft tick *Ornithodoros moubata*. *J. Immunol.* 174 2084–2091. 10.4049/jimmunol.174.4.2084 15699138

[B176] Nuttall G. H. F. Strickland C. (1908). On the presence of an anticoagulin in the salivary glands and intestines of *Argas persicus*. *Parasitology* 1 302–310. 10.1017/s0031182000003590

[B177] Nuttall G. H. F. Warburton C. Cooper W. F. Robinson L. E. (1908). *Ticks. A Monograph of the Ixodoidae. Part I: The Argasidae.* Cambridge: Cambridge University Press 10.1017/s0031182000003590

[B178] Nuttall P. A. (2019). Wonders of tick saliva. *Ticks Tick Borne Dis.* 10 470–481. 10.1016/j.ttbdis.2018.11.005 30459085

[B179] Nuttall P. A. Trimnell A. R. Kazimirova M. Labuda M. (2006). Exposed and concealed antigens as vaccine targets for controlling ticks and tick-borne diseases. *Parasite Immunol.* 28 155–163. 10.1111/j.1365-3024.2006.00806.x 16542317

[B180] Oleaga-Pérez A. Pérez-Sánchez R. Astigarraga A. Encinas-Grandes A. (1994). Detection of pig farms with *Ornithodoros erraticus* by pig serology. Elimination of non-specific reactions by carbohydrate epitopes of salivary antigens. *Vet. Parasitol.* 52 97–111. 10.1016/0304-4017(94)90040-x 8030193

[B181] Oliveira C. J. Anatriello E. de Miranda-Santos I. K. Francischetti I. M. Sá-Nunes A. Ferreira B. R. (2013). Proteome of *Rhipicephalus sanguineus* tick saliva induced by the secretagogues pilocarpine and dopamine. *Ticks Tick Borne Dis.* 4 469–477. 10.1016/j.ttbdis.2013.05.001 24029695 PMC3917560

[B182] Packham M. A. Rand M. L. (2011). Historical perspective on ADP-induced platelet activation. *Purinergic. Signal.* 7 283–292. 10.1007/s11302-011-9227-x 21484086 PMC3166988

[B183] Paesen G. C. Adams P. L. Harlos K. Nuttall P. A. Stuart D. I. (1999). Tick histamine-binding proteins: isolation, cloning, and three-dimensional structure. *Mol. Cell* 3 661–671. 10.1016/s1097-2765(00)80359-7 10360182

[B184] Paesen G. C. Adams P. L. Nuttall P. A. Stuart D. L. (2000). Tick histamine-binding proteins: lipocalins with a second binding cavity. *Biochim. Biophys. Acta* 1482 92–101. 10.1016/s0167-4838(00)00168-0 11058751

[B185] Paesen G. C. Siebold C. Dallas M. L. Peers C. Harlos C. Nuttall P. A. (2009). An ion-channel modulator from the saliva of the brown ear tick has a highly modified Kunitz/BPTI structure. *J. Mol. Biol.* 389 734–747. 10.1016/j.jmb.2009.04.045 19394347

[B186] Paesen G. C. Siebold C. Harlos K. Peacey M. F. Nuttall P. A. Stuart D. I. (2007). A tick protein with a modified Kunitz fold inhibits human tryptase. *J. Mol. Biol.* 368 1172–1186. 10.1016/j.jmb.2007.03.011 17391695

[B187] Parizi L. F. Githaka N. W. Acevedo C. Benavides U. Seixas A. Logullo C. (2013). Sequence characterization and immunogenicity of cystatins from the cattle tick Rhipicephalus (Boophilus) microplus. *Ticks Tick Borne Dis.* 4 492–499. 10.1016/j.ttbdis.2013.06.005 24035585

[B188] Perner J. Kropáčková S. Kopáček P. Ribeiro J. M. C. (2018). Sialome diversity of ticks revealed by RNAseq of single tick salivary glands. *PLoS Negl. Trop. Dis.* 12:e0006410. 10.1371/journal.pntd.0006410 29652888 PMC5919021

[B189] Pervaiz S. Brew K. (1987). Homology and structure-function correlations between alpha 1-acid glycoprotein and serum retinol-binding protein and its relatives. *FASEB J.* 1 209–214. 10.1096/fasebj.1.3.3622999 3622999

[B190] Petersen L. J. (1997). Quantitative measurement of extracellular histamine concentrations in intact human skin in vivo by the microdialysis technique: methodological aspects. *Allergy* 52 547–555. 10.1111/j.1398-9995.1997.tb02598.x 9201366

[B191] Pichu S. Ribeiro J. M. Mather T. N. Francischetti I. M. (2014). Purification of a serine protease and evidence for a protein C activator from the saliva of the tick, *Ixodes scapularis*. *Toxicon* 77 32–39. 10.1016/j.toxicon.2013.10.025 24184517 PMC3877196

[B192] Pienaar R. Neitz A. W. H. Mans B. J. (2018). Tick paralysis: solving an Enigma. *Vet. Sci.* 5:53. 10.3390/vetsci5020053 29757990 PMC6024606

[B193] Platt E. J. Wehrly K. Kuhmann S. E. Chesebro B. Kabat D. (1998). Effects of CCR5 and CD4 cell surface concentrations on infections by macrophagetropic isolates of human immunodeficiency virus type 1. *J. Virol.* 72 2855–2864. 9525605 10.1128/jvi.72.4.2855-2864.1998 PMC109730

[B194] Preston S. G. Majtán J. Kouremenou C. Rysnik O. Burger L. F. Cabezas Cruz A. (2013). Novel immunomodulators from hard ticks selectively reprogramme human dendritic cell responses. *PLoS Pathog.* 9:e1003450. 10.1371/journal.ppat.1003450 23825947 PMC3695081

[B195] Prevot P. P. Adam B. Boudjeltia K. Z. Brossard M. Lins L. Cauchie P. (2006). Anti-hemostatic effects of a serpin from the saliva of the tick *Ixodes ricinus*. *J. Biol. Chem.* 281 26361–26369. 10.1074/jbc.m604197200 16672226

[B196] Prevot P. P. Beschin A. Lins L. Beaufays J. Grosjean A. Bruys L. (2009). Exosites mediate the anti-inflammatory effects of a multifunctional serpin from the saliva of the tick *Ixodes ricinus*. *FEBS J.* 276 3235–3246. 10.1111/j.1742-4658.2009.07038.x 19438720

[B197] Prié S. Guillemette G. Boulay G. Borgeat P. Sirois P. (1995). Leukotriene C4 receptors on guinea pig tracheocytes. *J. Pharmacol. Exp. Ther.* 275 312–318. 7562565

[B198] Radulović,ŽM. Mulenga A. (2017). Heparan sulfate/heparin glycosaminoglycan binding alters inhibitory profile and enhances anticoagulant function of conserved *Amblyomma americanum* tick saliva serpin 19. *Insect Biochem. Mol. Biol.* 80 1–10. 10.1016/j.ibmb.2016.11.002 27845251 PMC5214524

[B199] Rangel C. K. Parizi L. F. Sabadin G. A. Costa E. P. Romeiro N. C. Isezaki M. (2017). Molecular and structural characterization of novel cystatins from the taiga tick *Ixodes persulcatus*. *Ticks Tick Borne Dis.* 8 432–441. 10.1016/j.ttbdis.2017.01.007 28174118

[B200] Rawal N. Pangburn M. (2001). Formation of high-affinity C5 convertases of the alternative pathway of complement. *J. Immunol.* 166 2635–2642. 10.4049/jimmunol.166.4.2635 11160326

[B201] Rawal N. Pangburn M. K. (2003). Formation of high affinity C5 convertase of the classical pathway of complement. *J. Biol. Chem.* 278 38476–38483. 10.1074/jbc.m307017200 12878586

[B202] Ribeiro J. M. (1987). *Ixodes dammini*: salivary anti-complement activity. *Exp. Parasitol.* 64 347–353. 10.1016/0014-4894(87)90046-4 3119364

[B203] Ribeiro J. M. Arcà B. (2009). From sialomes to the sialoverse: an insight into salivary potion of blood-feeding insects. *Adv. In. Insect Physiol.* 37 59–118. 10.1371/journal.pone.0044612 23049752 PMC3458046

[B204] Ribeiro J. M. Evans P. M. MacSwain J. L. Sauer J. (1992). Amblyomma americanum: characterization of salivary prostaglandins E2 and F2 alpha by RP-HPLC/bioassay and gas chromatography-mass spectrometry. *Exp. Parasitol.* 74 112–116. 10.1016/0014-4894(92)90145-z 1730268

[B205] Ribeiro J. M. Mather T. N. (1998). *Ixodes scapularis*: salivary kininase activity is a metallo dipeptidyl carboxypeptidase. *Exp. Parasitol.* 89 213–221. 10.1006/expr.1998.4296 9635445

[B206] Ribeiro J. M. C. (1995). How ticks make a living. *Parasitol. Today* 11 91–93. 10.1016/0169-4758(95)80162-6 15275359

[B207] Ribeiro J. M. C. Endris T. M. Endris R. (1991). Saliva of the soft tick *Ornithodoros moubata*, contains anti-platelet and apyrase activity. *Comp. Biochem. Physiol.* 100 109–112. 10.1016/0300-9629(91)90190-n 1682082

[B208] Ribeiro J. M. C. Makoul G. Levine J. Robinson D. Spielman A. (1985). Antihemostatic, antiinflammatory, and immunosuppressive properties of the saliva of a tick *Ixodes dammini*. *J. Exp. Med.* 161 332–344. 10.1084/jem.161.2.332 2982989 PMC2187567

[B209] Ribeiro J. M. C. Spielman A. (1986). Ixodes dammini: salivary anaphylatoxin inactivating activity. *Exp. Parasitol.* 62 292–297. 10.1016/0014-4894(86)90034-2 3743719

[B210] Riek R. F. (1957). Studies on the reactions of animals to infestation with ticks. *Aust. J. Agric. Res.* 8 215–223.

[B211] Riek R. F. (1959). Studies on the reactions of animals to infestation with ticks. *Aust. J. Agric. Res.* 10 604–613.

[B212] Rodriguez-Valle M. Moolhuijzen P. Barrero R. A. Ong C. T. Busch G. Karbanowicz T. (2017). Transcriptome and toxin family analysis of the paralysis tick, *Ixodes holocyclus*. *Int. J. Parasitol.* 48 71–82. 10.1016/j.ijpara.2017.07.007 28989068

[B213] Rodriguez-Valle M. Xu T. Kurscheid S. Lew-Tabor A. E. (2015). *Rhipicephalus microplus* serine protease inhibitor family: annotation, expression and functional characterisation assessment. *Parasit. Vectors* 8:7. 10.1186/s13071-014-0605-4 25564202 PMC4322644

[B214] Ross I. C. (1926). An experimental study of tick paralysis in Australia. *Parasitology* 18 410–429. 10.1186/s13071-018-3061-8 30157914 PMC6116354

[B215] Roversi P. Johnson S. Preston S. G. Nunn M. A. Paesen G. C. Austyn J. M. (2017). Structural basis of cholesterol binding by a novel clade of dendritic cell modulators from ticks. *Sci. Rep.* 7:16057. 10.1038/s41598-017-16413-2 29167574 PMC5700055

[B216] Roversi P. Lissina O. Johnson S. Ahmat N. Paesen G. C. Ploss K. (2007). The structure of OMCI, a novel lipocalin inhibitor of the complement system. *J. Mol. Biol.* 369 784–793. 10.1016/j.jmb.2007.03.064 17445829 PMC2724154

[B217] Roversi P. Ryffel B. Togbe D. Maillet I. Teixeira M. Ahmat N. (2013). Bifunctional lipocalin ameliorates murine immune complex-induced acute lung injury. *J. Biol. Chem.* 288 18789–18802. 10.1074/jbc.M112.420331 23625922 PMC3696655

[B218] Sabbatani L. (1899). Fermento anticoagulante del’ *Ixodes ricinus*. *Arch. Ital. Biol.* 31 37–53.

[B219] Sadik C. D. Miyabe Y. Sezin T. Luster A. D. (2018). The critical role of C5a as an initiator of neutrophil-mediated autoimmune inflammation of the joint and skin. *Semin. Immunol.* 37 21–29. 10.1016/j.smim.2018.03.002 29602515

[B220] Salát J. Paesen G. C. Rezácová P. Kotsyfakis M. Kovárová Z. Sanda M. (2010). Crystal structure and functional characterization of an immunomodulatory salivary cystatin from the soft tick *Ornithodoros moubata*. *Biochem. J.* 429 103–112. 10.1042/BJ20100280 20545626 PMC3523712

[B221] Sangamnatdej S. Paesen G. C. Slovak M. Nuttall P. A. (2002). A high affinity serotonin- and histamine-binding lipocalin from tick saliva. *Insect Mol. Biol.* 11 79–86. 10.1046/j.0962-1075.2001.00311.x 11841505

[B222] Sant’Anna Azzolini S. Sasaki S. D. Torquato R. J. Andreotti R. Andreotti E. Tanaka A. S. (2003). *Rhipicephalus sanguineus* trypsin inhibitors present in the tick larvae: isolation, characterization, and partial primary structure determination. *Arch. Biochem. Biophys.* 417 176–182. 10.1016/s0003-9861(03)00344-8 12941299

[B223] Sasagawa M. Satoh T. Takemoto A. Hasegawa T. Suzuki E. Arakawa M. (1994). Blood levels of leukotrienes (LTC4, D4, E4, B4) in asthmatic patients during attack and remission. *Arerugi* 43 28–36. 8147706

[B224] Schroeder H. Daix V. Gillet L. Renauld J. C. Vanderplasschen A. (2007). The paralogous salivary anti-complement proteins IRAC I and IRAC II encoded by *Ixodes ricinus* ticks have broad and complementary inhibitory activities against the complement of different host species. *Microbes Infect.* 9 247–250. 10.1016/j.micinf.2006.10.020 17223370

[B225] Schuijt T. J. Bakhtiari K. Daffre S. Deponte K. Wielders S. J. Marquart J. A. (2013). Factor Xa activation of factor V is of paramount importance in initiating the coagulation system: lessons from a tick salivary protein. *Circulation* 128 254–266. 10.1161/CIRCULATIONAHA.113.003191 23817575 PMC3826089

[B226] Schuijt T. J. Narasimhan S. Daffre S. DePonte K. Hovius J. W. Van’t Veer C. (2011). Identification and characterization of *Ixodes scapularis* antigens that elicit tick immunity using yeast surface display. *PLoS One* 6:e15926. 10.1371/journal.pone.0015926 21246036 PMC3016337

[B227] Schwarz A. Tenzer S. Hackenberg M. Erhart J. Gerhold-Ay A. Mazur J. (2014). A systems level analysis reveals transcriptomic and proteomic complexity in *Ixodes ricinus* midgut and salivary glands during early attachment and feeding. *Mol. Cell. Proteomics* 13 2725–2735. 10.1074/mcp.M114.039289 25048707 PMC4188998

[B228] Scott E. (1921). Hume and Hovell’s journey to Port Phillip. *Roy. Aust. Hist. Soc.* 7 289–380.

[B229] Sears D. W. Thompson S. E. Saxon S. R. (2007). Reversible ligand binding reactions: why do biochemistry students have trouble connecting the dots? *Biochem. Mol. Biol. Educ.* 35 105–118. 10.1002/bmb.29 21591070

[B230] Singh K. Davies G. Alenazi Y. Eaton J. R. O. Kawamura A. Bhattacharya S. (2017). Yeast surface display identifies a family of evasins from ticks with novel polyvalent CC chemokine-binding activities. *Sci. Rep.* 7:4267. 10.1038/s41598-017-04378-1 28655871 PMC5487423

[B231] Smith R. D. Engdahl A. L. Dunbar J. B. Jr. Carlson H. A. (2012). Biophysical limits of protein-ligand binding. *J. Chem. Inf. Model.* 52 2098–2106. 10.1021/ci200612f 22713103 PMC3428497

[B232] Soares C. A. Lima C. M. Dolan M. C. Piesman J. Beard C. B. Zeidner N. S. (2005). Capillary feeding of specific dsRNA induces silencing of the Isac gene in nymphal *Ixodes scapularis* ticks. *Insect Mol. Biol.* 14 443–452. 10.1111/j.1365-2583.2005.00575.x 16033437

[B233] Soares T. S. Watanabe R. M. Tanaka-Azevedo A. M. Torquato R. J. Lu S. Figueiredo A. C. (2012). Expression and functional characterization of Boophilin, a thrombin inhibitor from *Rhipicephalus* (Boophilus) microplus midgut. *Vet. Parasitol.* 187 521–528. 10.1016/j.vetpar.2012.01.027 22341830

[B234] Sokol C. L. Luster A. D. (2015). The chemokine system in innate immunity. *Cold Spring Harb. Perspect. Biol.* 7:a016303. 10.1101/cshperspect.a016303 25635046 PMC4448619

[B235] Sonenshine D. E. (2004). Pheromones and other semiochemicals of ticks and their use in tick control. *Parasitology* 129 S405–S425. 15938521 10.1017/s003118200400486x

[B236] Stutzer C. Mans B. J. Gaspar A. R. Neitz A. W. Maritz-Olivier C. (2009). *Ornithodoros savignyi*: soft tick apyrase belongs to the 5’-nucleotidase family. *Exp. Parasitol.* 122 318–327. 10.1016/j.exppara.2009.04.007 19393241

[B237] Tanaka A. S. Andreotti R. Gomes A. Torquato R. J. Sampaio M. U. Sampaio C. A. (1999). A double headed serine proteinase inhibitor–human plasma kallikrein and elastase inhibitor–from *Boophilus microplus* larvae. *Immunopharmacology* 45 171–177. 10.1016/s0162-3109(99)00074-0 10615008

[B238] Tang J. Fang Y. Han Y. Bai X. Yan X. Zhang Y. (2015). YY-39, a tick anti-thrombosis peptide containing RGD domain. *Peptides* 68 99–104. 10.1016/j.peptides.2014.08.008 25152502

[B239] Tatchell R. J. (1967). A modified method for obtaining tick oral secretion. *J. Parasitol.* 53 1106–1107. 6062065

[B240] Tatchell R. J. Moorhouse D. E. (1968). The feeding processes of the cattle tick *Boophilus microplus* (Canestrini). II. The sequence of host-tissue changes. *Parasitology* 58 441–459. 5740485 10.1017/s0031182000069468

[B241] Tirloni L. Islam M. S. Kim T. K. Diedrich J. K. Yates J. R. III Pinto A. F. (2015). Saliva from nymph and adult females of *Haemaphysalis longicornis*: a proteomic study. *Parasit. Vectors* 8:338. 10.1186/s13071-015-0918-y 26104117 PMC4484640

[B242] Tirloni L. Reck J. Terra R. M. Martins J. R. Mulenga A. Sherman N. E. (2014). Proteomic analysis of cattle tick *Rhipicephalus* (Boophilus) microplus saliva: a comparison between partially and fully engorged females. *PLoS One* 9:e94831. 10.1371/journal.pone.0094831 24762651 PMC3998978

[B243] Tyson K. Elkins C. Patterson H. Fikrig E. de Silva A. (2007). Biochemical and functional characterization of Salp20, an *Ixodes scapularis* tick salivary protein that inhibits the complement pathway. *Insect Mol. Biol.* 16 469–479. 10.1111/j.1365-2583.2007.00742.x 17651236

[B244] Tyson K. R. Elkins C. de Silva A. M. (2008). A novel mechanism of complement inhibition unmasked by a tick salivary protein that binds to properdin. *J. Immunol.* 180 3964–3968. 10.4049/jimmunol.180.6.3964 18322205

[B245] Uhlír J. Grubhoffer L. Borskı I. Dusbábek F. (1994). Antigens and glycoproteins of larvae, nymphs and adults of the tick *Ixodes ricinus*. *Med. Vet. Entomol.* 8 141–150. 10.1111/j.1365-2915.1994.tb00154.x 8025322

[B246] Valdés J. J. Cabezas-Cruz A. Sima R. Butterill P. T. Růžek D. Nuttall P. A. (2016). Substrate prediction of *Ixodes ricinus* salivary lipocalins differentially expressed during *Borrelia afzelii* infection. *Sci. Rep.* 6:32372. 10.1038/srep32372 27584086 PMC5008119

[B247] Valdés J. J. Schwarz A. Cabeza de Vaca I. Calvo E. Pedra J. H. Guallar V. (2013). Tryptogalinin is a tick Kunitz serine protease inhibitor with a unique intrinsic disorder. *PLoS One* 8:e62562. 10.1371/journal.pone.0062562 23658744 PMC3643938

[B248] Valenzuela J. G. Charlab R. Mather T. N. Ribeiro J. M. (2000). Purification, cloning, and expression of a novel salivary anticomplement protein from the tick, *Ixodes scapularis*. *J. Biol. Chem.* 275 18717–18723. 10.1074/jbc.m001486200 10749868

[B249] van de Locht A. Stubbs M. T. Bode W. Friedrich T. Bollschweiler C. Höffken W. (1996). The ornithodorin-thrombin crystal structure, a key to the TAP enigma? *EMBO J.* 15 6011–6017. 10.1002/j.1460-2075.1996.tb00989.x 8947023 PMC452422

[B250] Viljoen G. J. Neitz A. W. H. Prozesky L. Bezuidenhout J. D. Vermeulen N. M. J. (1985). Purification and properties of tick egg toxic proteins. *Insect Biochem.* 15 475–482. 10.1016/0020-1790(85)90060-5

[B251] von Siebold C. T. H. Stannius H. (1854). *Comparative Anatomy.* London: Trubner and Company.

[B252] Wagner C. L. Mascelli M. A. Neblock D. S. Weisman H. F. Coller B. S. Jordan R. E. (1996). Analysis of GPIIb/IIIa receptor number by quantification of 7E3 binding to human platelets. *Blood* 88 907–914. 8704248

[B253] Wang H. Kaufman W. R. Cui W. W. Nuttall P. A. (2001b). Molecular individuality and adaptation of the tick *Rhipicephalus appendiculatus* in changed feeding environments. *Med. Vet. Entomol.* 15 403–412. 10.1046/j.0269-283x.2001.00328.x 11776459

[B254] Wang H. Hails R. S. Cui W. W. Nuttall P. A. (2001a). Feeding aggregation of the tick *Rhipicephalus appendiculatus* (Ixodidae): benefits and costs in the contest with host responses. *Parasitology* 123 447–453. 11719955 10.1017/s0031182001008654

[B255] Wang H. Nuttall P. A. (1994). Comparison of the proteins in salivary glands, saliva and haemolymph of *Rhipicephalus appendiculatus* female ticks during feeding. *Parasitology* 109 517–523. 7794318 10.1017/s003118200008077x

[B256] Wang H. Paesen G. C. Nuttall P. A. Barbour A. G. (1998). Male ticks help their mates to feed. *Nature* 391 753–754. 10.1038/35773 9486641

[B257] Wang X. Coons L. B. Taylor D. B. Stevens S. E. Jr. Gartner T. K. (1996). Variabilin, a novel RGD-containing antagonist of glycoprotein IIb-IIIa and platelet aggregation inhibitor from the hard tick *Dermacentor variabilis*. *J. Biol. Chem.* 271 17785–17790. 10.1074/jbc.271.30.17785 8663513

[B258] Wang Y. Yu X. Cao J. Zhou Y. Gong H. Zhang H. (2015). Characterization of a secreted cystatin from the tick *Rhipicephalus haemaphysaloides*. *Exp. Appl. Acarol.* 67 289–298. 10.1007/s10493-015-9946-8 26188856

[B259] Watts S. W. Morrison S. F. Davis R. P. Barman S. M. (2012). Serotonin and blood pressure regulation. *Pharmacol. Rev.* 64 359–388. 10.1124/pr.111.004697 22407614 PMC3310484

[B260] Waxman L. Connolly T. M. (1993). Isolation of an inhibitor selective for collagen-stimulated platelet aggregation from the soft tick *Ornithodoros moubata*. *J. Biol. Chem.* 268 5445–5449. 8449906

[B261] Waxman L. Smith D. E. Arcuri K. E. Vlasuk G. P. (1990). Tick anticoagulant peptide (TAP) is a novel inhibitor of blood coagulation factor Xa. *Science* 248 593–596. 10.1126/science.2333510 2333510

[B262] Whisstock J. C. Silverman G. A. Bird P. I. Bottomley S. P. Kaiserman D. Luke C. J. (2010). Serpins flex their muscle: II. Structural insights into target peptidase recognition, polymerization, and transport functions. *J. Biol. Chem.* 285 24307–24312. 10.1074/jbc.R110.141408 20498368 PMC2915666

[B263] Wiegner R. Chakraborty S. Huber-Lang M. (2016). Complement-coagulation crosstalk on cellular and artificial surfaces. *Immunobiology* 221 1073–1079. 10.1016/j.imbio.2016.06.005 27371975

[B264] Wikel S. (2017). “Arthropod modulation of wound healing. in arthropod vector,” in *Controller of Disease Transmission* Vol. 2 eds Wikel S. K. Aksoy S. Dimopoulos G. (Amsterdam: Elsevier) 31–50. 10.1016/b978-0-12-805360-7.00003-4

[B265] Xu A. Rodriguez-Valle M. (2016). Effective inhibition of thrombin by *Rhipicephalus microplus* serpin-15 (RmS-15) obtained in the yeast *Pichia pastoris*. *Ticks Tick Borne Dis.* 7 180–187. 10.1016/j.ttbdis.2015.09.007 26530984

[B266] Xu X. L. Cheng T. Y. Yang H. (2016). Enolase, a plasminogen receptor isolated from salivary gland transcriptome of the ixodid tick *Haemaphysalis flava*. *Parasitol. Res.* 115 1955–1964. 10.1007/s00436-016-4938-0 26822735

[B267] Yokomizo T. (2015). Two distinct leukotriene B4 receptors, BLT1 and BLT2. *J. Biochem.* 157 65–71. 10.1093/jb/mvu078 25480980

[B268] Zhu K. Bowman A. S. Brigham D. L. Essenberg R. C. Dillwith J. W. Sauer J. R. (1997a). Isolation and characterization of Americanin, a specific inhibitor of thrombin, from the salivary glands of the lone star tick *Amblyomma americanum* (L.). *Exp. Parasitol.* 87 30–38. 10.1006/expr.1997.4175 9287955

[B269] Zhu K. Sauer J. R. Bowman A. S. Dillwith J. W. (1997b). Identification and characterization of anticoagulant activities in the saliva of the lone star tick, *Amblyomma americanum* (L.). *J. Parasitol.* 83 38–43. 9057694

